# Hunt–Vitell’s General Theory of Marketing Ethics Predicts “Attitude-Behaviour” Gap in Pro-environmental Domain

**DOI:** 10.3389/fpsyg.2022.732661

**Published:** 2022-03-02

**Authors:** Laura Zaikauskaitė, Gemma Butler, Nurul F. S. Helmi, Charlotte L. Robinson, Luke Treglown, Dimitrios Tsivrikos, Joseph T. Devlin

**Affiliations:** ^1^Department of Clinical, Educational and Health Psychology, University College London, London, United Kingdom; ^2^Thomas International Limited, Marlow, United Kingdom; ^3^Department of Experimental Psychology, University College London, London, United Kingdom

**Keywords:** moral judgements, pro-environmental behaviour, climate change, attitude – behaviour gap, General Theory of Marketing Ethics, Hunt–Vitell

## Abstract

The inconsistency between pro-environmental attitudes and behaviours, known as the “attitude-behaviour” gap, is exceptionally pronounced in scenarios associated with “green” choice. The current literature offers numerous explanations for the reasons behind the “attitude-behaviour” gap, however, the generalisability of these explanations is complex. In addition, the answer to the question of whether the gap occurs between attitudes and intentions, or intentions and behaviours is also unknown. In this study, we propose the moral dimension as a generalisable driver of the “attitude-behaviour” gap and investigate its effectiveness in predicting attitudes, pro-environmental intentions and subsequent behaviours. We do so by using Hunt–Vitell’s moral philosophy-based framework of ethical decision-making, which conceptualises morality as the central decision-making parameter. The results from 557 US MTurk participants revealed that the manipulation of moral dimensions, specifically deontology and teleology, impacted ethical evaluation of presented dilemmas, however, failed to translate into subsequent intentions and behaviours. This finding suggests (i) that the moral dimension has an effect in shaping attitudes toward environmental issues, and (ii) that gap occurs between attitudes and intentions rather than intentions and behaviours. Further investigation of what strengthens and/or overrides the effects of the moral dimension would help understand the reasons why moral attitudes do not always translate into subsequent intentions and behaviours in the pro-environmental domain.

## Introduction

In recent years, climate change has come into focus leading consumers to make “green” choices that could potentially reverse their impact upon the environment. This new reality created within the consumer’s mindset the notion that a “green” choice is an ethical choice, since “green” purchasing protects the environment (Lu et al., 2015). Indeed, scholars have conceptualised pro-environmental behaviour as a form of moral behaviour (Markowitz and Shariff, 2012) and defined an ethical consumer as one who makes conscious and deliberate choices to follow certain consumption patterns due to moral beliefs (Crane and Matten, 2004). However, studies attempting to investigate environmental behaviour from a moral perspective have not provided clear results (Markowitz and Shariff, 2012), which could then be generalised to the overall environmental domain. Importantly, existing literature lacks studies explaining how moral dimension relates to attitudes, intentions, and behaviours, which are the three main components of many expectancy-value or rational choice models, such as Value-Belief-Norm theory (VBN, Stern, 2000) or the Theory of Planned Behaviour (TPB, Ajzen, 1991). Currently, the precise reasons why positive attitude toward pro-environmental behaviour does not often translate to subsequent actions are unclear (Grimmer and Miles, 2017) and lead to a common debate of the drivers of the “attitude-behaviour” gap (Auger and Devinney, 2007; Carrington et al., 2010).

Hence, we aim to further how the moral component impacts environmental attitudes, intentions, and subsequent behaviours. Specifically, we aim to answer the questions (i) whether the “attitude-behaviour” gap occurs between attitude and intention or intention and behaviour, and (ii) whether the moral component could account for the “attitude-behaviour” gap. We have chosen to meet our aims by addressing the “attitude-behaviour” gap issue in the moral philosophy-based General Theory of Marketing Ethics (GTME; Hunt and Vitell, 1986, 2006) and consequent framework (Hunt and Vasquez-Parraga, 1993), introduced by Hunt and Vitell (1986). We believe Hunt–Vitell’s model is the most suitable because it conceptualises the notion of a moral element as a central parameter rather than an additional layer to the overall decision-making process. This is to say that we will be able to investigate the relationship between morality, attitudes, intentions and behaviours; given the robust effects over a number of studies involving ethical issues (Hunt, 2016). To our knowledge, none of the pro-environmental studies which have employed Hunt–Vitell’s framework (e.g., Lu et al., 2015; Arikan and Jiang, 2017; Nimri et al., 2021) incorporated both intention and behaviour-based measures to the model, making it difficult to predict whether the “attitude-behaviour” gap occurs between attitudes and intentions or intentions and behaviours. Below we present the review of the non-exhaustive list of studies, which highlight the key issues with existing “attitude-behaviour” gap research and portray the potential of a moral dimension in defining pro-environmental decision-making.

### Investigations of the “Attitude-Behaviour” Gap in the Environmental Domain

Numerous attempts have been made to investigate the determinants of the “attitude-behaviour” gap in environmental decision-making, however, the factors driving “attitude-behaviour” inconsistencies, as well as the question of whether the gap occurs between the attitudes and behaviours, or between the intentions and behaviours, remain unspecified (Kollmuss and Agyeman, 2002). This is because the issue has been studied using a wide range of theories and methods, which led to the findings being behaviour – or context-specific, thus mostly ungeneralisable to the overall domain. For instance, Wiederhold and Martinez (2018) investigated the factors influencing decision-making in the green apparel industry by conducting interviews and analysing them according to the Grounded Theory approach (Goulding, 2005; Glaser and Strauss, 2017), which led to identifying the effects of price, availability, knowledge, transparency, image, inertia and consumption habits. In contrast, Dhir et al. (2021) examined green apparel purchasing using the Knowledge-Attitude-Behaviour model (Kallgren and Wood, 1986) and Attitude-Behaviour-Context Theory (Guagnano et al., 1995) and found the “attitude-behaviour” gap to be impacted by environmental knowledge, green trust, and environmental concern. The impact of trust has also been investigated by Taufique et al. (2017), who conducted a quantitative study in the context of eco-label knowledge. Similarly, to Dhir et al. (2021) and Wu et al. (2021) applied Attitude-Behaviour-Context Theory to the longitudinal investigation of the determinants leading to the inconsistencies between pro-environmental intentions made during holiday versus further pro-environmental behaviours at home. Likewise, the reasons for the engagement with unsustainable behaviours during the holiday period were qualitatively investigated by Juvan and Dolnicar (2014), who grounded their findings in Cognitive Dissonance Theory (Festinger, 1957). Alternatively, De Pelsmacker et al. (2005) and Schäufele and Hamm (2018) examined fair trade coffee and organic wine purchasing using cluster analysis rather than any pre-determined theory and found that “attitude-behaviour” alignment within some of the identified clusters was dependent on certain sociodemographic characteristics (e.g., gender, age, education) and other factors, such as idealism, which signifies the possible underlying impact of moral beliefs. However, no generalisable conclusions on what drives the “attitude-behaviour” gap in the environmental domain, and where exactly the gap occurs, were outlined. Therefore, the question of whether there is a clear, generalisable determinant of the “attitude-behaviour” gap and how this determinant fits within existing psychological and philosophical theories is yet to be answered. The potentially generalisable dimension which could be disrupting the proposed translation from positive attitudes to subsequent pro-environmental behaviours is that of morality, broadly defined as the understanding of what is a “right” or “wrong” action to take in a given situation (Paton, 1948; Hauser, 2006). In fact, it’s important to point out that morality is a context dependent construct (Komarova Loureiro et al., 2016). This means that the understanding of what is a moral thing to do is fluid, and changes according to specific settings (such as, e.g., history, culture, race, economy, geography, etc.). Therefore, we believe that conceptualising moral dimension as a central factor (Hunt and Vitell, 1986, 2006), rather than as an external predictor of chosen behaviours (Kaiser and Scheuthle, 2003; Kaiser et al., 2005; Hübner and Kaiser, 2006; Kaiser, 2006; Chan and Bishop, 2013) could lead to more accurate results in assessing the actual effects of morality. It could also explain why the effects of moral dimension differ across the studies in different geographic locations, and could suggest the answers to the questions why the effects of common variables such as price, knowledge, habits, etc. predict “attitude-behaviour” gap in different ways across the studies (see Yaprak and Prince, 2019 for a review). Therefore, exploring the effects of the moral dimension could provide more generalisable answers to the drivers of the “attitude-behaviour” gap in a pro-environmental domain.

### Morality and Pro-environmental Behaviours: Empirical Studies

Of the theoretical approaches that were applied to explore the relationship between morality and environmental behaviour, perhaps the most significant are the ones that have studied environmental morality in a form of altruism, empathic capacity to relate to nature, or have conceptualised moral norms to behave pro-environmentally as a matter of social responsibility (Batson, 1991; Batson and Shaw, 1991; Thøgersen, 2006; Berenguer, 2010). This led to (i) investigating developmental perspectives using Hoffman’s (1983) and Kohlberg’s (1984) theories, (ii) building in anthropocentric, biospheric, ecocentric, egoistic or non-environmental value orientations to experimental designs (Eckersley, 1992; Merchant, 1992; Thompson and Barton, 1994; Kortenkamp and Moore, 2001), (iii) scrutinising moral rhetoric and moral identity in political and consumer behaviour contexts (Feinberg and Willer, 2013; Wolsko et al., 2016; Hahn and Garrett, 2017; Wolsko, 2017); or (iv) studying the predictive capacity of unmodified or modified expectancy-value/rational-choice models (Ajzen, 1991; Stern, 2000). Despite that, the explanation of how morality translates from attitudes to pro-environmental behaviours is not yet complete.

From a psychological point of view, one’s perception of a moral norm results from moral cognition and moral effect (Gibbs, 1991). Two major developmental perspectives suggest that moral development starts in the early childhood, when the understanding of moral norms and values is being progressively constructed over time (Cognitive Moral Development Theory; Kohlberg, 1984) and is being transmitted from society to a child (Moral Socialisation Theory; Hoffman, 1983). Both scholars suggest that moral development involves cognitive and affective components. However, Kohlberg’s (1984) focuses on cognition, highlighting the importance of abstract reasoning based on an understanding of the consequences of one’s behaviour and the context. In contrast, Hoffman’s (1983) focuses on the importance of empathetic feelings for moral development. According to Hoffman (1983), moral norm develops when others point out the consequences of hurtful behaviour and how it makes the hurt one feel. Therefore, these two theoretical accounts fulfil one another by bridging the connection between moral reasoning and empathy (Lazarus, 1984; Zajonc, 1984; Sigel, 1986).

Some authors have applied these developmental theories to explore pro-environmental morality. For example, Littledyke (2004) conducted qualitative research with Year 1–6 school children and found that younger children had little to no understanding of the term “environment,” and performed pro-environmental actions because of their “obedience to authority” and “conformity to rules” rather than because of the sense of morality. In contrast, children in school years 5/6 demonstrated the capacity for the moral consideration to the environment, which is in line with Kohlberg’s (1984) Cognitive Moral Development theory suggesting that capacity for moral reason develops over time. However, most of the Year 5/6 children did not relate moral awareness to their own actions impacting the environment, therefore raising the question of whether moral awareness will translate into subsequent actions in the later development.

Karpiak and Baril (2008) extended the use of Kohlberg’s (1984) theory to anthropocentric and ecocentric views. According to anthropocentric attitudes, human beings are the only ones who have moral significance; other living organisms, ecosystems, populations, species, or land have value in relation to how useful they are to humans, rather than have value on their own. Therefore, humans are dominant over nature (Eckersley, 1992; Evernden and Evernden, 1992). Such anthropocentric attitudes were challenged by the newer branches of environmental ethics such as ecocentrism (Purser et al., 1995). According to ecocentric views, moral significance should be attributed to all living beings, ecosystems, natural wilderness and Earth itself; nature and its beings have value independent of human needs (Rodman, 1977). Indeed, Karpiak and Baril (2008) found that ecocentrism was related to principled moral reasoning (the most advanced level of moral development; Kohlberg, 1984), whereas anthropocentrism was not. However, the measures of how such moral reasoning about the environment would further translate into pro-environmental behaviours were not included in their study, leaving the relationship between moral attitudes and behaviours undefined.

The relationship between anthropocentric, ecocentric and non-environmental^1^ attitudes and moral reasoning patterns in pre-service science teachers has been studied by Tuncay et al. (2011). Similarly, to the aforementioned study by Karpiak and Baril (2008), Tuncay et al. (2011) have also found the connection between positive environmental attitudes and ecocentrism, but not anthropocentrism or non-environmentalism, suggesting that positive attitudes may result from moral reasoning about the state of nature itself, rather than the focus on how environmental problems affect humans.

The consideration of how biocentric attitudes (i.e., the idea that nature has moral standing independent of humans) impact environmental behaviour has been included in the qualitative study by Severson and Kahn (2010), who have found that the majority of 7–12-year-old children demonstrated biocentric reasoning by judging pesticide use in farming as “wrong,” suggesting that moral consideration for nature can develop even earlier than previously established in the literature (Kahn, 1999). Despite that, the majority of the same children have also accepted the use of pesticides in the orchards where they lived. Severson and Kahn (2010) suggested this attitude-behaviour inconsistency could be due to the fact that some children believed pesticides were safe to humans or justified their use by associating them to jobs for their families, bringing up the issues of financial security.

In economics, the standard assumption is that individuals act to maximise utility, in other words, the benefit to themselves (Mill, 1962; Turaga et al., 2010). According to a famous model of homo economicus, individual’s behaviour is based on rational self-interest or strictly egoistic motivations (Mill, 1871). This assumption, however, is contradictory to account for behaviours of voluntary charitable giving, pro-environmental acts, etc., therefore has been challenged by many economic theorists who have noted that the model can’t be complete without incorporating more socially sophisticated elements, such as values, altruism, social status or social norms (note: social norm does not need to have moral nor rational component, e.g., it may simply be a matter of fashion, etc.; Anderson, 2000). Therefore, economists have introduced the concept of ‘impure altruism’, defined as the motivation to contribute to the public good, albeit for egoistic reasons (e.g., to derive “warm glow” benefit, such as prestige or social approval; Andreoni, 1990). Brekke et al. (2003) have proposed and contradict the framework by suggesting that utility from the act of giving may not necessarily stand from pure self-interest but from moral reason to maintain a self-image of a socially responsible person. In addition, their model recognises that a person’s willingness to act according to one’s “morally ideal” image is limited by the costs of contributing to that effort, be it costs of convenience, time, finance, etc. This model has been applied by Weaver (1996), who showed that environmentally friendly agricultural practices depend on individual characteristics, such as values, beliefs, and attitudes, therefore categorising people into selfish hedonists (those who derive utility from profits), egoistic hedonists (those who care about profits and the ward glow benefits of contributing to public good), altruists (those who derive utility from profits and the aggregate quantity of public good), and imperfect altruists (those who derive utility from profits, their own contribution, and the aggregate quantity of the public good) allowed to explain why certain types of individuals will (not) respond to profitable agricultural practices. Similarly, Chouinard et al. (2008) have suggested that individual preferences depend on active utility function, specifically, either personal interest (ego-utility) or moral/social interest (s-utility). However, individuals with preferences for both might not seek to maximise either of the components but search for the compromise. Based on this idea, Nyborg et al. (2006) have used evolutionary game theory to propose that the equilibrium in pro-environmental consumption could only be achieved if either everyone or no one would buy environmental products, therefore providing background why other than moral functions might become more active in motivating (pro-)environmental behaviours.

A different line of studies has focused on exploring the effects of morality using so-called expectancy-value or rational choice models, such as Schwartz’s (1977) and its later modification to VBN Theory (Stern, 2000), or the Theory of Reasoned Action (Fishbein and Ajzen, 1977) and its later modification to TPB (Ajzen, 1991). However, many studies incorporating morality in these models either assessed the relationship between morality and attitudes, morality and intention, or morality and behaviour, but not all the four variables at once, making it difficult to track how and whether moral dimension translates through attitudes to intentions to behaviours. For example, a study by Chen (2015) have used the VBN theory of environmentalism, which incorporates the measures for egocentric, altruistic, and biocentric values, and proposes their chain effects on intention and/or behaviour through the New Ecological Paradigm (Catton and Dunlap, 1980), Awareness of Consequences, and Ascription of Responsibility. In their study where only the behaviour rather than intention and behaviour were measured, Chen (2015) has found that egocentric values had the least impact, whereas both altruistic and biocentric values had approximately five times more weight in predicting pro-environmental behaviour of Taiwanese consumers. In contrast, Steg et al. (2005) have found that biocentric values were positively related to intention, egoistic values had a negative impact and altruistic values had no impact on intention to accept energy policies, again suggesting that some effects of morality may be context-specific.

Many pro-environmental studies that have built moral norms into the TPB propose that intention and behaviour is a result of attitudes, subjective norms (the degree to which the behaviour is believed to be a “norm”), and perceived behavioural control (the degree to which a person can execute the behaviour; Ajzen, 1991) have either included intention or behaviour, but not both measures into the model. This makes it difficult to identify whether the “attitude-behaviour” gap occurs between attitudes and intention, or intention and behaviour. Furthermore, the few studies that have included all three measures and the items for morality have resulted in contrasting findings. For example, the study by Chan and Bishop (2013) has revealed that significant correlation coefficients between moral norms and attitudes (*r* = 0.60) or moral norms and intention (*r* = 0.50) were of similar weight, whereas correlation coefficient predicting association between moral norms and recycling behaviour was much smaller (*r* = 0.35), suggesting that moral norms were less associated with actual recycling behaviours. Interestingly, Donald et al. (2014) have also included moral norms into their study and, similarly to the study by Chan and Bishop (2013), have found a significant correlation with attitudes and the correlation coefficient was of similar strength (*r* = 0.70). However, the associations between moral norms and both intention (*r* = 0.47) and transport use behaviour (*r* = 0.36) were much weaker, suggesting a gap between the attitudes and intention rather than intention and behaviour. In contrast, the study by Pakpour et al. (2014) included moral obligation rather than moral norms and found the significant correlation coefficient between attitudes to be much smaller (*r* = 0.36) than in the aforementioned studies by Chan and Bishop (2013) (*r* = 0.60) and Donald et al. (2014) (*r* = 0.70). However, the significant correlation coefficients between moral obligation and both intention and recycling behaviour were stronger and of the same size (*r* = 0.54), suggesting that the moral dimension had less impact in influencing attitudes but more impact in driving both intention and behaviours, which is contradictory to the “attitude-behaviour” theory which suggests that the strength of positive attitudes influence subsequent behaviours (Armitage and Christian, 2003). The current state of literature, which incorporates morality, attitude, intention, and behaviour measures into the TPB (e.g., Kaiser and Scheuthle, 2003; Tonglet et al., 2004; Bezzina and Dimech, 2011; Wan et al., 2014; Graham-Rowe et al., 2015; Xu et al., 2017; Mak et al., 2018; Strydom, 2018) makes it difficult to rule out the reasons why the association of the moral element varies across the studies, as well as answer the question of whether the gap occurs between attitudes and intentions, or intentions and behaviours.

In summary, the effects of the moral dimension have been documented in a number of pro-environmental behaviour studies. Whereas some have identified other than moral factors as more influential in certain situations and contexts, the overall state of literature supports the positive relationship between morality and pro-environmental behaviour. The challenge with understanding how morality impacts pro-environmental behaviour, however, lies in the way existing quantitative and qualitative studies were conducted, making it difficult to better define how morality fits within the “attitude-behaviour” gap literature.

### Theoretical Framework: Hunt–Vitell’s General Theory of Marketing Ethics

To be able to systematically investigate the key research questions (i) whether “attitude-behaviour” gap occurs between attitude and intention or intention and behaviour, and (ii) whether moral component could account for “attitude-behaviour” gap, it is necessary to render the study into a well-known theory or model. Reviewed studies suggest the idea that pro-environmental behaviour results from rational rather than an irrational thought process, making expectancy-value or rational choice models such as VBN (Stern, 2000) or the TPB (Ajzen, 1991) a good fit for the proposed research. However, one shortcoming of the TPB is that its original version does not assume moral element as a central driver of attitudes, intentions and behaviours, and thus requires additional modifications. In contrast, the VBN model proposes value orientations, such as biocentrism, altruism, egoism as the starting point which drives further intentions and behaviours; however, it falls short in the extent to which different contexts could be tested in a single study, therefore lacks sufficient fit with proposed research questions.

A common way to test the effects of contexts in a single study is by incorporating different environmental scenarios into ethical dilemmas (e.g., Zeidler and Schafer, 1984; Axelrod, 1994; Požarnik, 1995; Flannery and May, 1999; Kortenkamp and Moore, 2001; Berenguer, 2010; Tuncay et al., 2011; Crumpei et al., 2014; Jia et al., 2017). The well-known framework that allows doing so is that of Hunt–Vitell’s model of ethical decision-making, which results from the GTME (Hunt and Vitell, 1986, 2006). Hunt–Vitell’s model suggests that the reasoning process is triggered once the individual recognises ethical aspects in a dilemma. According to the model, the perception of ethical aspects results from an individual’s moral code (Hunt, 2016), making morality a central aspect of the model. Here, moral code is composed of deontological philosophy, which conceptualises moral norms in terms of duties and obligations (i.e., rightness and wrongness of behaviour itself), and teleological philosophy^2^, which seeks to maximise the best consequences for the given situation (i.e., how many good vs. bad outcomes will the decision generate) and thus allows immoral means to achieve the greatest good. In the model, the effects of deontological and teleological dimensions are being assessed by manipulating ethical versus unethical actions of the actor (deontology) and positive versus negative consequences of the behaviour (teleology) and asking a participant to judge the ethicality of a given dilemma. Such ethical judgement is proposed to translate into intentions and subsequent behaviours. Thus, we believe it fits well with investigating proposed research questions.

Attempts to apply Hunt–Vitell’s model demonstrated its consistency in predicting the relationship between personal moral philosophy and behaviour, although this was mostly done in organisational (Hunt and Vasquez-Parraga, 1993; Burns and Kiecker, 1995; Mengüç, 1998; Donoho et al., 2001; Hunt and Laverie, 2004) or general consumer settings (Mayo and Marks, 1990; Vitell et al., 2001). Few of the studies, however, have also applied Hunt–Vitell’s theory to investigate green consumption choices (Lu et al., 2015; Arikan and Jiang, 2017; Nimri et al., 2021), albeit without incorporating the dilemmas into the model. For example, Lu et al. (2015) have used a modified version of Hunt–Vitell’s model to factors, rather than deontological and teleological philosophies. Specifically, the authors have considered cultural factors of individualism vs. collectivism and conceptualise consumers’ ethical beliefs as a function of cultural and personal personal factors of attitude toward business and loyalty proneness. The results revealed the positive relationship between high ethical awareness and intention to buy green products, suggesting that participants who perceived recycling and/or other pro-environmental behaviours as ethically acceptable were more willing to reconsider questionable but legal business practices and make decisions according to their ethical standards. A qualitative application of Hunt–Vitell’s model to study consumers’ ethical beliefs toward dining in green restaurants have been conducted by Nimri et al. (2021), who have identified deontological and teleological evaluations in shaping ethical beliefs, and subsequent links to dining in green restaurants. Specifically, deontological evaluations were reflected by participants’ beliefs of personal responsibility for environmental wellbeing, leading to perceiving green restaurant choice as “the right thing.” Teleological evaluations were reflected by considering the consequences of participants’ choice of restaurant on various stakeholders, such as their own body, family members, community, physical environment, and future generations. Neither of the studies employing Hunt–Vitell’s model to investigate pro-environmental issues, however, have considered assessing the relationship between attitudes, intentions and behaviours, leaving the answers to the questions (i) whether “attitude-behaviour” gap occurs between attitude and intention or intention and behaviour, and (ii) whether a moral component could account for the “attitude-behaviour” gap unanswered. Therefore, based on the reviewed literature, we hypothesise:

**H1**: Participants rely on deontological framing when forming ethical evaluations.
**H2**: Participants rely on teleological framing when forming ethical evaluations.
**H3**: Participants rely on ethical evaluation when forming (a) intention and (b) behaviour.

## Materials and Methods

### Participants and Procedures

The data from 692 US participants was collected using Amazon’s Mechanical Turk. The study was divided into two parts and took approx.—3 and 10 min to complete, respectively. Participants were paid $0.35 and $1.80 for completing each piece. University College London Ethics Committee granted ethics approval for this study, and all participants gave online consent. The results were computed using IBM SPSS v.26 and AMOS v.27.

### Design and Stimuli

The study consisted of a 2-deontological framing (unethical vs. ethical behaviour) × 2-teleological framing (negative vs. positive consequences) between-participants design. To reduce the effects of the context, we have created 10 real-life dilemmas that resemble each item of the environmental behaviours scale (Huang, 2016). For example, the dilemma resembling the first behaviour item, “Recycle newspapers, plastics, cans and glass” incorporated recycling and explicitly stated its consequences to the current climate change situation (Table 1). The manipulation of deontological and teleological frames was done by creating four different endings for each of the dilemmas. For example, dilemmas 1A and 1B depicted the unethical behaviour of the main character, whereas dilemmas 1C and 1D depicted the ethical behaviour of the main character (deontological manipulations). Similarly, dilemmas 1B and 1D depicted negative consequences to the environment, whereas dilemmas 1A and 1C depicted positive consequences to the environment (teleological manipulations).

**TABLE 1:** Example of environmental dilemma.

Dilemma 1: Recycling newspapers, plastics, cans and glass.	
**Situation:** John and his teammates are in charge of the props for the school’s theatre production. Some of the materials used to make the props were glass, cans newspapers and plastics. John has decided to not keep any of the props after they were used due to lack of storage, but there are only 10 min left to clear the props before the premises closes. **Climate Impact:** John knows that not recycling will lead to factories constantly producing new materials, which requires depletion of natural resources. Recycling helps save natural resources, energy, and reduce the carbon emission pollution that would come from producing new materials every time.	

**Manipulations**	

**(1A) Unethical behaviour:** Given the situation, John decides to throw away the props since they are running out of time. **Circumstances:** However, his teammates decide to bring the props home so they could recycle them the next day. **Positive Consequences:** The teammates’ decision means that the props will be recycled and will not end up in the landfill.	**(1C) Ethical behaviour:** Given the situation, John decides to tell his teammates to bring the props home due to shortage of time, and plans to meet up the next day to sort out the props based on recycling category. **Circumstances:** n/a **Positive Consequences:** John’s decision means that the props will be recycled and will not end up in the landfill.
**(1B) Unethical behaviour:** Given the situation, John decides to throw away the props since they are running out of time. **Circumstances**. n/a **Negative Consequences:** John’s decision means that the props will not be recycled and will therefore end up in a landfill.	**(1D) Ethical behaviour**: Given the situation, John decides to tell his teammates to bring the props home due to shortage of time, and plans to meet up the next day to sort out the props based on recycling category. **Circumstances**. However, his teammates think it’s too much work and simply throw the props away without recycling. **Negative Consequences:** The teammates’ decision means that the props will not be recycled and will therefore end up in a landfill.

In contrast to the previous design by Hunt and Vasquez-Parraga (1993) or Mengüç (1998), our study incorporated 10 rather than 2 dilemmas because we expected this to reduce context-induced measurement errors. Specifically, it is possible that some people place more importance on certain pro-environmental behaviours, while other behaviours may receive less attention because individuals do not believe them to be of high significance to improving the climate change situation. The perception of which behaviours are more important than others is dependent upon the individual. Therefore, we believed that increasing variability in the contexts would allow obtaining more generalisable results. As in previous designs by Hunt and Vasquez-Parraga (1993) or by Mengüç (1998), each participant was shown 2 random dilemmas of the same condition (e.g., 2×As, 2×Bs, 2×Cs, or 2×Ds), which ensured that the respondents were blind to the manipulations.

### Measures

#### Pro-environmental Behaviours

Ten pro-environmental behaviour items measuring everyday pro-environmental behaviours such as recycling, electricity, transportation etc., were adapted from Huang’s (2016) study. The frequency of performing presented behaviours was measured on a 7-point Likert scale (e.g., “Recycle newspapers, plastics and glass,” “Compost kitchen waste”; 1-never, 7-every time). Their Cronbach’s α was 0.81 (Huang, 2016).

#### Intention

Following the technique to transform behaviour scale into intension scale (Kaiser and Scheuthle, 2003; Kaiser, 2006), we have asked participants to rate 10 pro-environmental behaviour items (Huang, 2016). To reduce the error of obtaining socially desirable responses, intention was measured on two 7-point semantic differential scales (“I intend to…”, (1) Unlikely – Likely, (2) Undetermined – Determined), resulting in two intention items per corresponding behaviour item.

#### Deontological and Teleological Manipulations

Following the coding procedures of Hunt and Vasquez-Parraga (1993), deontological manipulation was treated as a dummy variable with 0 for unethical behaviour and 1 for ethical behaviour conditions. Similarly, teleological manipulation of experimental conditions was treated as a dummy variable with 0 for negative outcomes and 1 for positive outcomes.

#### Ethical Evaluation

Ethical evaluation of presented dilemma was assessed with two items (“I consider John’s actions to be very ethical,” “Most people would consider John’s actions to be very ethical”), adapted from Vitell et al. (2001) and measured on a 7-point Likert scale (1-strongly disagree, 7-strongly agree).

#### Attention Check Items

Two attention check items were included in the study. After responding to the Ethical evaluation scale, participants were presented with a new page and were asked to write 2–3 sentences summarising the key details of the scenario that had been presented on the previous page. This helped ensure that participants were humans rather than bots (Moss and Litman, 2018) and that they had read the presented dilemma (Paas et al., 2018; Aguinis et al., 2020).

### Procedures

The online survey was launched using the Qualtrics survey platform and set such that all questions on the page needed to be answered before moving on to the next page with questions. The participants completed the study in the web browser. To minimise social desirability bias, which is often present in pro-environmental behaviour studies (Chao and Lam, 2009; Vesley and Klöckner, 2020), we have divided the survey into two parts, which were conducted 5 weeks apart. Part 1 of the survey consisted of the pro-environmental behaviour scale (Huang, 2016) and demographics. Part 2 of the survey consisted of 20 intention items, 2 randomly presented dilemmas and 4 ethical evaluations items (2 per presented dilemma). The data from Part 1 and Part 2 of the study were merged using anonymous response ID, and the merged dataset was utilised for further analyses.

## Results

### Analysis Procedures and Rationale

First, we ran exploratory factor analysis to check whether questionnaire items loaded on correct factors, indicating their suitability for further multivariate analysis. Second, we ran correlational analyses to assess the strength and directionality of relationships between the study variables. Third, we ran confirmatory factor analysis (CFA) to test the data fit and adjust it for the structural model. As the data was not normally distributed, maximum likelihood with robust standard errors was used for parameter estimation. Based upon Kline’s (2005) recommendations, the following fit indices were applied: the χ^2^/df ratio, Root Mean Square Error of Approximation (RMSEA), Standardised Root Mean Residual (SRMR), Comparative Fit Index (CFI), and the Tucker-Lewis Index (TLI). Fourth, we ran an ANOVA to assess the differences between experimental conditions. Fifth, we have tested the hypotheses using path analyses.

### Data Cleaning

We have discarded the participants who failed to provide correct answers to the attention check items. Specifically, all answers were reviewed to ensure that the participants had read and understood the presented dilemmas. Seventy-nine participants failed to answer one or both attention check questions meaningfully and thus were removed from the dataset. In addition, to ensure that adequate attention to behaviour and both sets of intention items was given, we have discarded the responses below the duration of 10 s per scale of 10 items. This resulted in removing additional 55 responses. The final sample consisted of 557 participants.

### Sample Demographics

The final sample consisted of 50% females and 49% males (1% preferred not to say). Most of the respondents were 25–34 (35%) and 35–49 (33%) years of age and were either single (36%) or married with children (33%). In addition, 50% of the participants were college graduates, 61% were in full-time employment, and 33% reported an annual household income level of $75,000 or more (Table 2).

**TABLE 2:** Sample demographics (*N* = 557).

Demographics	Item	*N*	%
Gender	Male	277	50
	Female	272	49
	Prefer not to say	8	1
Age	18–24	21	3
	25–34	215	39
	35–49	183	33
	50–64	107	19
	65 and above	31	6
Marital Status	Single (never married)	199	36
	Married (no children)	70	12
	Married (with children)	185	33
	Domestic partnership	40	7
	Divorced	11	2
	Widowed	4	1
	Separated	48	9
Education	High school or less	47	9
	Some college	113	20
	Undergraduate	0	0
	College graduate	278	50
	Post collegiate	118	21
	None of the above	0	0
Employment Status	Full time	341	61
	Part time	60	11
	Self-employed	60	11
	Unemployed	40	7
	Retired	35	6
	Student	12	2
	Other	9	2
Household Income	Less than $9,999	16	3
	$10,000–$19,999	38	7
	$20,000–$29,999	46	8
	$30,000–39,999	64	11
	$40,000–$49,999	63	11
	$50,000–74,999	148	27
	$75,000 or more	182	33

### Exploratory Factor Analysis

To validate the measures, 4 ethical evaluations, 20 intentions, and 10 behaviour items were selected for the exploratory factor analysis (principal components with a Promax rotation; Hair et al., 1998). Four behaviour and eight corresponding intention items were dropped because they did not load on correct factors. The final set of measures consisted of 4 ethical evaluations, 6 behaviour, and 12 corresponding intention items, which loaded on 7 factors with a total variance of 78%. Unexpectedly, each of the remaining behaviour and corresponding intention items loaded together on six “intention-behaviour” factors rather than 2 separate “intention” and “behaviour” factors. This natural factor solution suggested that behaviour items could not be statistically distinguished from the corresponding intention items, which would suggest that there is no gap between intention and behaviour.

The final factor solution consisted of one factor composed of four ethical evaluation items and six factors, each composed of one behaviour and two corresponding intention items. All seven factors had internal consistency estimates above 0.60, yielded eigenvalues greater than 1.0, communalities and factor loadings greater than 0.50, which is well above the limit of acceptability (Aiken et al., 1991). The results presented in Table 3 show the final seven-factor solution, items, consistency estimates, eigenvalues, variance, factor loadings, and communality statistics.

**TABLE 3:** The results of exploratory factor analysis.

Factors and items	Factor loadings	Communalities
*Factor 1: Ethical Evaluation. Cronbach’s α = 0.87, Eigenvalue = 5.857, Variance = 26.62%*		
I consider actions shown in the first scenario to be very ethical	0.865	0.756
Most people would consider actions shown in the first scenario to be very ethical	0.868	0.763
I consider actions shown in the second scenario to be very ethical	0.845	0.725
Most people would consider actions shown in the second scenario to be very ethical	0.827	0.695
*Factor 2: Intention and Behaviour – Recycling. Cronbach’s α = 0.87, Eigenvalue = 2.912, Variance = 13.24%*		
Likelihood of recycling newspapers, plastics, can sand glass (intention)	0.897	0.868
Determination to recycle newspapers, plastics, can sand glass (intention)	0.851	0.863
Frequency of recycling newspapers, plastics, can sand glass (behaviour)	0.882	0.755
*Factor 3: Intention and Behaviour – Composting. Cronbach’s α = 0.90, Eigenvalue = 2.340, Variance = 10.64%*		
Likelihood of composting kitchen waste (intention)	0.883	0.795
Determination to compost kitchen waste (intention)	0.836	0.817
Frequency of composting kitchen waste (behaviour)	0.946	0.829
*Factor 4: Intention and Behaviour – Transportation. Cronbach’s α = 0.85, Eigenvalue = 1.935, Variance = 8.80%*		
Likelihood of reducing driving and walk, bike or use public transport instead (intention)	0.928	0.858
Determination to reduce driving and walk, bike or use public transport instead (intention)	0.853	0.806
Frequency of reducing driving and walk, bike or use public transport instead (behaviour)	0.838	0.704
*Factor 5: Intention and Behaviour – Meat Consumpt. Cronbach’s α = 0.89, Eigenvalue = 1.531, Variance = 6.96%*		
Likelihood of eating less meat and more vegetables (intention)	0.936	0.883
Determination to eat less meat and more vegetables (intention)	0.944	0.873
Frequency of eating less meat and more vegetables (behaviour)	0.803	0.695
*Factor 6: Intention and Behaviour – Energy Use. Cronbach’s α = 0.82, Eigenvalue = 1.375, Variance = 6.25%*		
Likelihood of buying energy-efficient appliances (intention)	0.915	0.850
Determination to buy energy-efficient appliances (intention)	0.938	0.856
Frequency of buying energy-efficient appliances (behaviour)	0.659	0.534
*Factor 7: Intention and Behaviour – Utensils. Cronbach’s α = 0.79, Eigenvalue = 1.173, Variance = 5.33%*		
Likelihood of bringing own utensils when eating out (intention)	0.901	0.823
Determination to bring own utensils when eating out (intention)	0.896	0.801
Frequency of bringing own utensils when eating out (behaviour)	0.674	0.574
Total variance = 78%		
KMO = 0.722		
χ^2^ = 8512.490		
df = 231		
Sig. = 0.000		

### Normality, Descriptive Statistics and Spearman Correlations

Shapiro–Wilk tests were run to test the assumption of normality. Results have revealed that the data was not normally distributed. Further investigation of univariate and multivariate outliers was carried out. Skewness and kurtosis indexes were less than 3 and 10, respectively, and Cook’s distances for all the variables were lower than 1 (varying from 0.000 to 0.265; Stevens, 1992; see Supplementary Table 1). Thus, the deviation from normality found was not considered severe (Kline, 2011). Therefore, it was still deemed appropriate to report the means as a measure of central tendency.

Descriptive statistics for the final sample analysed are summarised in Table 4. We have used Spearman’s rho to compute the correlation matrix for the further CFA and path analysis because this non-parametric measure of association makes no distributional assumptions. This avoids distorting the distribution if there is a reason to believe these characteristics are representative of the underlying population (Norman and Streiner, 2008).

**TABLE 4:** Descriptive statistics, skewness and kurtosis per four experimental conditions.

	Variable	Mean	*SD*	Skewness	Kurtosis
	**Dilemmas A (Unethical, Positive), *N* = 141**				
1	Ethical Evaluation	4.75	1.35	–0.49	0.00
2	Intention and Behaviour – Recycling	5.70	1.39	–1.45	1.82
3	Intention and Behaviour – Composting	3.69	2.07	0.20	0.41
4	Intention and Behaviour – Transportation	4.29	1.76	–0.27	–1.00
5	Intention and Behaviour – Meat Consumpt.	4.26	1.72	–0.12	–0.88
6	Intention and Behaviour – Energy Use	5.44	1.21	–1.09	1.42
7	Intention and Behaviour – Utensils	2.60	1.69	0.95	–0.05
	**Dilemmas B (Unethical, Negative), *N* = 139**				
1	Ethical Evaluation	4.26	1.45	–0.06	–0.46
2	Intention and Behaviour – Recycling	5.67	1.56	–1.38	1.07
3	Intention and Behaviour – Composting	3.31	2.10	0.48	–1.22
4	Intention and Behaviour – Transportation	3.99	1.74	–0.17	–0.97
5	Intention and Behaviour – Meat Consumpt.	4.27	1.87	–0.18	–1.10
6	Intention and Behaviour – Energy Use	5.42	1.31	–0.94	0.81
7	Intention and Behaviour – Utensils	2.26	1.53	1.23	0.63
	**Dilemmas C (Ethical, Positive), *N* = 136**				
1	Ethical Evaluation	5.75	1.11	–0.88	0.58
2	Intention and Behaviour – Recycling	5.63	1.41	–1.28	1.04
3	Intention and Behaviour – Composting	3.32	2.01	0.47	–1.16
4	Intention and Behaviour – Transportation	4.09	1.81	–0.22	–1.09
5	Intention and Behaviour – Meat Consumpt.	4.11	1.85	–0.17	–1.12
6	Intention and Behaviour – Energy Use	5.33	1.32	–0.89	0.33
7	Intention and Behaviour – Utensils	2.32	1.56	1.08	0.13
	**Dilemmas D (Ethical, Negative), *N* = 141**				
1	Ethical Evaluation	5.51	1.02	–0.25	–0.96
2	Intention and Behaviour – Recycling	5.71	1.48	–1.35	1.05
3	Intention and Behaviour – Composting	3.33	1.95	0.53	–1.01
4	Intention and Behaviour – Transportation	4.10	1.67	0.10	–0.95
5	Intention and Behaviour – Meat Consumpt.	4.37	1.72	–0.27	–0.93
6	Intention and Behaviour – Energy Use	5.54	1.29	–1.01	0.90
7	Intention and Behaviour – Utensils	2.41	1.66	1.17	0.41

Next, to test the strength and directionality of the relationships between variables, we ran Spearman’s correlations because this test does not assume the parametric distribution of data (see Tables 5A–D). The results revealed that most of the correlations between ethical evaluation and “intention- behaviour” factors were not significant. Transportation was the only variable that significantly correlated in three conditions (*r*_A_ = 0.17, *p*_A_ = 0.039; *r*_B_ = –0.29, *p*_B_ = 0.000; *r*_D_ = 0.25, *p*_D_ = 0.003), while meat consumption (*r*_D_ = 0.19, *p*_D_ = 0.027), energy use (*r*_C_ = 0.22, *p*_C_ = 0.012), and utensils (*r*_B_ = –0.22, *p*_B_ = 0.010) significantly correlated in only one of the conditions. Some significant correlations between “intention-behaviour” factors were also observed. This could be because certain participants may be performing some but not all of the pro-environmental behaviours, therefore may not place the same importance on every “intention-behaviour” case. In fact, this has been statistically reflected in the exploratory factor analysis, which has revealed that it would be statistically incorrect to merge all 10 behaviour items into one latent “behaviour” variable. Overall, results suggest that (i) each pro-environmental behaviour needs to be treated as a separate factor and that (ii) there is no gap between intention and behaviour.

**TABLE 5:** Spearman’s coefficients and *p* values for intercorrelations among study variables, for dilemmas **(A–D)**.

	Variable	1	2	3	4	5	6	7

**A**	**Dilemmas A (Unethical, Positive)**							
1	Ethical Evaluation	1						
	*p*							
2	Intention and Behaviour – Recycling	–0.15	1					
	*p*	0.079						
3	Intention and Behaviour – Composting	0.11	0.26**	1				
	*p*	0.195	0.002					
4	Intention and Behaviour – Transportation	0.17*	–0.19*	0.33*	1			
	*p*	0.039	0.025	0.000				
5	Intention and Behaviour – Meat Consumpt.	0.06	0.32***	0.22*	0.26**	1		
	*p*	0.467	0.000	0.01	0.002			
6	Intention and Behaviour – Energy Use	–0.07	0.22**	0.29**	0.23**	0.33***	1	
	*p*	0.408	0.008	0.001	0.007	0.000		
7	Intention and Behaviour – Utensils	0.05	0.05	0.50***	0.333***	0.38***	0.29**	1
	*p*	0.527	0.561	0.000	0.000	0.000	0.001	

**B**	**Dilemmas B (Unethical, Negative)**							

1	Ethical Evaluation	1						
	*p*							
2	Intention and Behaviour – Recycling	–0.11	1					
	*p*	0.19						
3	Intention and Behaviour – Composting	–0.13	0.21*	1				
	*p*	0.12	0.01					
4	Intention and Behaviour – Transportation	–0.29***	0.24**	0.33***	1			
	*p*	0.000	0.005	0.000				
5	Intention and Behaviour – Meat Consumpt.	–0.07	0.19*	0.31***	0.46***	1		
	*p*	0.42	0.025	0.000	0.000			
6	Intention and Behaviour – Energy Use	–0.16	0.35***	0.27**	0.20*	0.17*	1	
	*p*	0.056	0.000	0.001	0.021	0.046		
7	Intention and Behaviour – Utensils	–0.22*	0.27**	0.42	0.38***	0.26**	0.10	1
	*p*	0.010	0.001	0.000	0.000	0.002	0.259	

**C**	**Dilemmas C (Ethical, Positive)**							

1	Ethical Evaluation	1						
	*p*							
2	Intention and Behaviour – Recycling	0.15	1					
	*p*	0.09						
3	Intention and Behaviour – Composting	0.08	0.17	1				
	*p*	0.343	0.050					
4	Intention and Behaviour – Transportation	0.13	0.305***	0.39***	1			
	*p*	0.129	0.000	0.000				
5	Intention and Behaviour – Meat Consumpt.	0.14	0.280**	0.43***	0.56***	1		
	*p*	0.101	0.001	0.000	0.000			
6	Intention and Behaviour – Energy Use	0.22*	0.48***	0.35***	0.16	0.08*	1	
	*p*	0.012	0.000	0.000	0.072	0.334		
7	Intention and Behaviour – Utensils	0.069	0.07	0.49**	0.36***	0.43***	0.014	1
	*p*	0.424	0.451	0.000	0.000	0.000	0.867	

**D**	**Dilemmas D (Ethical, Negative)**							

1	Ethical Evaluation	1						
	*p*							
2	Intention and Behaviour – Recycling	0.21	1					
	*p*	0.02						
3	Intention and Behaviour – Composting	0.08	0.17	1				
	*p*	0.321	0.051					
4	Intention and Behaviour – Transportation	0.25**	0.23**	0.30***	1			
	*p*	0.003	0.005	0.000				
5	Intention and Behaviour – Meat Consumpt.	0.19*	0.31***	0.26**	0.33***	1		
	*p*	0.027	0.000	0.002	0.000			
6	Intention and Behaviour – Energy Use	0.11	0.47***	0.18*	0.22*	0.32***	1	
	*p*	0.181	0.000	0.035	0.010	0.000		
7	Intention and Behaviour – Utensils	0.09	0.16	0.50***	0.29***	0.41***	0.19*	1
	*p*	0.28	0.07	0.000	0.000	0.000	0.024	

*p < 0.05, **p < 0.01, ***p < 0.001.

### Confirmatory Factor Analysis

**FIGURE 1:**
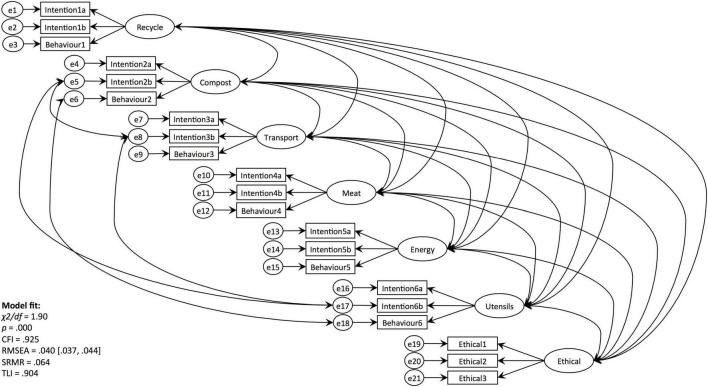
CFA: modified 7-factor model.

Seven-factor CFA was subjected to 4 experimental conditions where all 4 ethical evaluation items, 6 behaviour and 12 corresponding intention items were specified to load onto their respective factors. This model lacked data fit along the criteria of CFI and TLI (Table 6). Closer inspection of the covariance table revealed that the 4th ethical evaluation item was inflating the Chi-square value the most. Therefore, it was deleted from the model (Kline, 2005). In addition, several error terms were covaried (Figure 1). The Modified seven-Factor Model fitted the data well across the criteria of χ^2^/*df* = 1.90; RMSEA = 0.040 [0.037,0.044; CI 95%], SRMR = 0.074; TLI = 0.904. The value for CFI = 0.925 did not reach the 0.950 threshold recommended by Hooper et al. (2008). However, all the other metrics suggested a good model fit. Therefore, we have deemed obtained CFI value to be satisfactory for our analysis. Similarly, Chi-square test for the Modified Seven-Factor Model was significant [χ^2^(652) = 1241.76; *p* = 0.000], which is not in line with the expectation (Table 6). However, it’s not uncommon for the models using large data samples to achieve significant rather than expected non-significant *p*-value (Kline, 2005); therefore, we considered our confirmatory factor model to fit the data adequately well across the overall criteria.

**TABLE 6:** Goodness of fit results for the models subjected to CFA and path analyses.

Fit Index	7-Factor Model, CFA	Modified 7-Factor Model, CFA	7-Factor Structural Model, Path analysis	Modified 7-Factor Structural Model, Path analysis	Goodness of Fit criterion
χ^2^	2167.06	1241.76	1518.47	456.35	n/a
Df	752	652	218	196	n/a
χ^2^/*df*	2.88	1.90	6.97	2.33	<3^a^
*P*	0.000	0.000	0.000	0.000	>0.05^c^
CFI	0.834	0.925	0.834	.967	≥0.95^b^
RMSEA	0.058 [0.055,0.061]	0.040 [0.037,0.044]	0.104 [0.099,0.109]	0.049 [0.043,0.055]	<0.05^d^
SRMR	0.074	0.064	0.172	0.085	<0.08^b^
TLI	0.796	0.904	.	0.957	>0.90^c^

*CFI, Comparative Fit Index; RMSEA, Root Mean Square Error of Approximation [95% CI]; SRMR, Standardised Root Mean Square Residual; TLI, Tucker–Lewis Index.*

*^a^Values recommended by van Dam (2015).*

*^b^Values recommended by Hooper et al. (2008).*

*^c^Values recommended by Hu and Bentler (1998).*

*^d^Values recommended by MacCallum et al. (1996).*

### Ethical Evaluation: ANOVA

**FIGURE 2:**
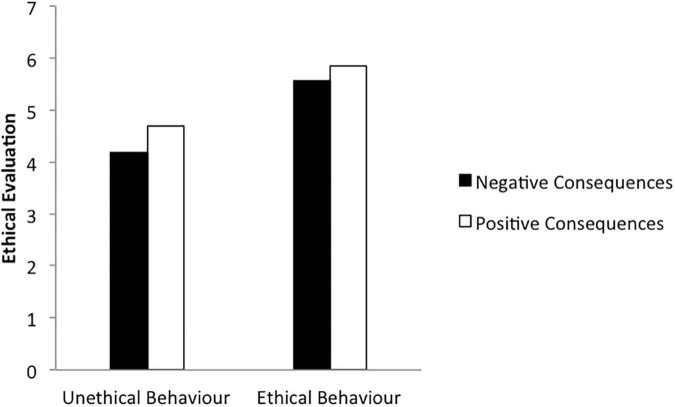
Ethical evaluation per deontological (unethical vs. ethical behaviour) and teleological (negative vs. positive consequences) frames.

The following analyses were performed to explore whether the manipulation of deontological and teleological framing impacted ethical evaluation of the actions presented in the dilemmas A–D. As expected, participants rated main character’s actions to be less ethical in both unethical (*M*_A_ = 4.70, *M*_B_ = 4.19) but not both ethical (*M*_C_ = 5.85, *M*_D_ = 5.57; Figure 2) conditions. Similarly, the actions presented in both negative conditions were rated to be less ethical than the actions presented in both positive conditions, respectively (*M*_B_ = 4.19 vs. *M*_A_ = 4.70; *M*_D_ = 5.57 vs. *M*_C_ = 5.85). ANOVA revealed that this manipulation has been statistically significant [*F*(3,553) = 49.58, *p* = 0.000, η_p_^2^ = 0.21]. In line with previous studies (Hunt and Vasquez-Parraga, 1993; Mengüç, 1998; Vitell et al., 2001), the effects of deontological dimension on ethical evaluation were stronger [*F*(1,556) = 135.35, *p* = 0.000, η_p_^2^ = 0.20] than the effects of teleological dimension [*F*(1,556) = 13.10, *p* = 0.000, η_p_^2^ = 0.02], suggesting that manipulation of the deontological frame (unethical vs. ethical behaviours) rather than teleological frame (negative vs. positive consequences) had a stronger impact on the difference in ethical evaluation ratings. The interaction of deontological and teleological dimensions was not observed [*F*(1,556) = 1.19, *p* = 0.28, η_p_^2^ = 0.00].

**FIGURE 3:**
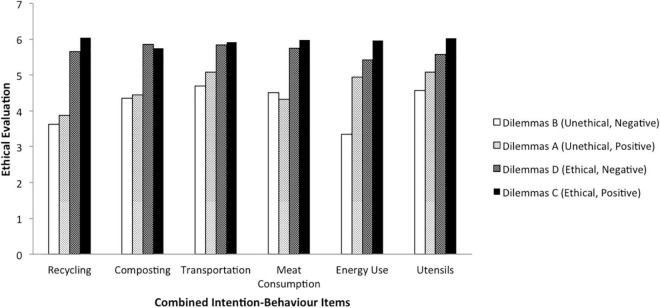
Ethical evaluation per “intention-behaviour” variables split according to deontological and teleological conditions.

We have further investigated whether the remaining six dilemmas held similar ethical evaluation patterns. Figure 3 below revealed that this had been the case for most of the dilemmas. The only two dilemmas that resulted in slightly different ethical evaluation patterns were those for composting and meat consumption.

Specifically, the effects of teleological manipulations on ethical evaluation in meat consumption dilemmas and the effects of deontological manipulations on ethical evaluation in composting dilemmas were the opposite from the rest of the ratings. The question of whether these inconsistencies are meaningful and are not due to the online research methodology requires further investigation. Overall, these two inconsistencies were very small, and thus we have not excluded these data from the further analyses.

### Structural Model

**FIGURE 4:**
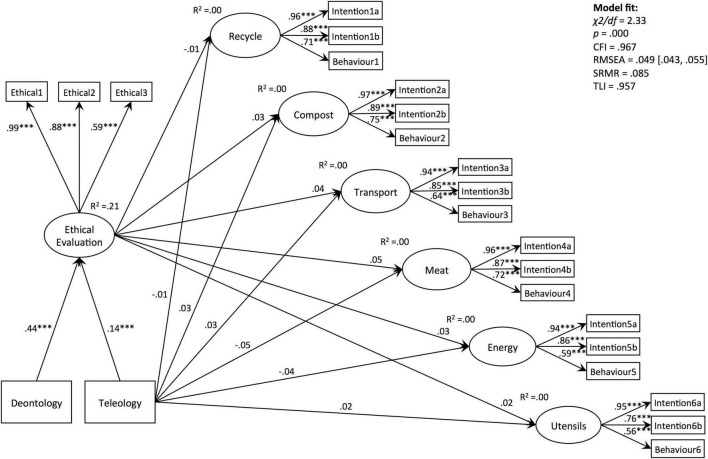
Modified 7-factor structural model (covariances not indicated). ***p < 0.001.

To test the hypotheses, dummy items indicating differences in deontological and teleological conditions, as well as 3 remaining ethical evaluations, 6 behaviour and 12 corresponding intention items, were retained for the path analyses and specified to load onto respective latent factors. All the indicators suggested poor model fit; therefore, several items were covaried. Modified Seven-Factor Structural Model fitted the data well across all the criteria (χ^2^/*df* = 2.33; CFI = 0.967; RMSEA = 0.049 [0.043,0.055; CI 95%]; SRMR = 0.085; TLI = 0.957), except for the fact that *p*-value for the Chi-square test was significant [χ^2^(196) = 456.35, *p* = 0.000; Figure 4 and Table 6; Kline, 2005].

Path analyses revealed significant effects of deontology and teleology on ethical evaluation (β*_*Deontology*_* = 0.44, *p* = 0.000; β*_*Teleology*_* = 0.14, *p* = 0.000), supporting hypotheses 1 and 2. In contrast, the effects of ethical evaluation on all “intention-behaviour” variables were not significant. Closer examination of indirect, direct, and total effects of deontology or teleology did not reveal statistical significance; therefore, hypothesis 3 proposing that ethical evaluation predicts “intention-behaviour” outcomes was not supported (Table 7).

**TABLE 7:** Hypotheses.

Hypothesis	Casual Path	Standartised Regression Weights	*p*	Conclusion
H1	Deontology → Ethical Evaluation	0.44	0.000	Supported
H2	Teleology → Ethical Evaluation	0.14	0.000	Supported
H3	Ethical Evaluation → Recycling Intention/Behaviour	–0.02	0.739	Not Supported
	Ethical Evaluation → Composting Intention/Behaviour	0.03	0.479	
	Ethical Evaluation → Transportation Intention/Behaviour	0.04	0.428	
	Ethical Evaluation → Consumption Intention/Behaviour	0.05	0.302	
	Ethical Evaluation → Energy Use Intention/Behaviour	0.03	0.535	
	Ethical Evaluation → Utensils Intention/Behaviour	0.02	0.689	

*p-Value significant above 0.05 threshold.*

### Summary of Results

Overall, the results suggest that deontological and teleological framing had a significant impact on ethical evaluation, meaning that participants’ ethical judgements were influenced by the manipulation of moral elements in the presented dilemmas, thus supporting hypotheses 1 and 2. This is consistent with the reviewed literature (Hunt and Vasquez-Parraga, 1993; Mengüç, 1998). Unexpectedly, exploratory factor analyses revealed that intention and behaviour items failed to load on two latent “intention” and “behaviour” factors. Instead, every behaviour and two corresponding intention items have loaded together, forming individual factors composed of three items. This natural solution of factor loadings suggested that (i) different individuals may be performing some but not all of the presented pro-environmental behaviours. Therefore, averaging all behaviour items into latent “intention” or “behaviour” variables may not lead to accurate results, and (ii) there was no statistical distinction between predicting intention and behaviour, indicating no gap between the two.

The further analysis demonstrated that the final structural model fit the data well. However, the paths from ethical evaluation to “intention-behaviour” factors were not significant, suggesting the gap between attitudes and “intention-behaviour” variables, thus rejecting hypothesis 3.

## General Discussion

The present study was designed to investigate the role of morality in predicting pro-environmental behaviours and its relationship to the “attitude-behaviour” gap. Specifically, we aimed to explore (i) whether the “attitude-behaviour” gap occurs between attitudes and intentions or intentions and behaviours and (ii) whether the moral component could account for the “attitude-behaviour” gap. In doing so, we have employed Hunt–Vitell’s ethical decision-making model (Hunt and Vasquez-Parraga, 1993; Mengüç, 1998), which is based on the GTME (Hunt and Vitell, 1986). This model differs from standard expectancy-value or rational choice models because it assumes morality as central parameter rather than an additional predictor of attitudes, intentions, and behaviours. To the best of our knowledge, no study to date has used Hunt–Vitell’s framework to assess the role of morality and its influence in predicting the “attitude-behaviour” gap. Therefore, our experiment serves as the first example of its effectiveness in addressing the aforementioned points.

As expected, we found that ethical evaluation of presented dilemmas was affected by manipulating moral dimensions, specifically, deontological (unethical vs. ethical behaviour) and teleological (negative vs. positive consequences) elements, thus supporting hypotheses 1 and 2. In line with the literature (Hunt and Vasquez-Parraga, 1993; Mengüç, 1998), deontological frames had a much stronger impact on ethical evaluation (β = 0.44; *p* = 0.000) than teleological frames (β = 0.14; *p* = 0.000). This showcased that participants paid attention (i) to the moral element and (ii) the type of moral element. In line with similar previous studies concerning ethics in organisational settings (Hunt and Vasquez-Parraga, 1993; Mengüç, 1998), the valence of behaviour itself (deontological frames) rather than the valence of the consequences of the behaviour (teleological frames) were considered more strongly, suggesting that the deontological dimension concerning “duty” and/or “obligation” influenced ethical evaluation more than the teleological dimension concerning the “calculation of the greatest good despite the behaviour itself being immoral.” One possible explanation for this finding is that the deontological dimension is considered to be concerned with justice and fairness, whereas teleological is considered to be concerned with perceived benefits and risks to see the expected results. Given the prominent topics of (in)justice, environmental racism, ecological debt, risk society, etc. (Marshall, 1999; Rasmussen, 2004; Warlenius et al., 2015), it is of no surprise that participants did not perceive teleological manipulations to be as worthy of their concern as deontological manipulations, thus relied on it much less during evaluating the ethicality of the dilemmas.

Unexpectedly, (i) our data suggested that it was statistically incorrect to average presented pro-environmental behaviours into one latent factor, and (ii) we also did not observe a statistically meaningful difference between the measurements of intentions vs. behaviours. The results would suggest (i) that participants did not perceive every pro-environmental behaviour as being of equal importance and (ii) that there was no gap between intentions and behaviours. In fact, we also failed to find the link between ethical evaluation and intentions-behaviours, thus rejecting hypothesis 3. As such, our findings provided evidence that the moral dimension had an impact on shaping attitudes but have not translated into intentions-behaviours. This is to say that the gap occurred between attitudes and intentions rather than intentions and behaviours.

Here, our findings raise further questions on why would some studies incorporating morality into expectancy value/rational choice models would observe stronger relationships between attitudes and intentions; whereas other ones would observe stronger relationships between intentions and behaviours (e.g., Chan and Bishop, 2013; Donald et al., 2014; Pakpour et al., 2014). One possible explanation rests upon the type of pro-environmental behaviour considered the given context, and the measurement of morality might have had an impact on obtained results. For example, the study by Chan and Bishop (2013), who found that moral norms associated with attitudes and intention were stronger when they were associated with behaviours, was done online using a sample of 271 Australian students (*M*_age_ = 24 years, *SD* = 7.31) and the pro-environmental behaviour of interest was the recycling of newspapers, glass and aluminium. In contrast, the study by Donald et al. (2014), who found that moral norms were more strongly associated with attitudes but not intentions and behaviours, was done either online or in-person using a sample of 827 United Kingdom drivers (*M*_age_ = 41 years; age range 17–78 years) and the pro-environmental behaviour of interest was transport use. Furthermore, the study by Pakpour et al. (2014), who included moral obligation rather than moral norms, found that morality was associated with intentions and behaviours more strongly than it was associated with attitudes was done in-person using a sample of 1782 Iranian participants (avg. participant age = 32 years; ±12.7 years) and the choice of pro-environmental behaviour was recycling. As a result, it is possible that lacking consistency in the way studies were conducted is the reason why they have produced contradicting effects of how morality relates to attitudes, intentions, and behaviours.

In fact, the environmental studies by Kaiser and Scheuthle (2003), Kaiser et al. (2005), Hübner and Kaiser (2006), Kaiser (2006), and Chan and Bishop (2013) have raised the question of how and where morality fits into the models that assume a direct intention-behaviour relationship. For example, it was debated whether moral norms should be added to the TPB as an additional variable alongside attitudes, subjective norms, and perceived behavioural control or should morality be treated as a predictor of attitudes themselves. Interestingly, some studies found that the addition of moral norms did not increase the predictive capacity of the TPB, suggesting the idea that morality is already reflected in the attitudes and there is no discriminant validity between the two constructs (Kaiser, 2006; Chan and Bishop, 2013). Indeed, according to the conceptualisation of Hunt–Vitell’s model, it would follow that moral dimension is already reflected in the attitudes, which are measured in the form of ethical evaluation. Therefore, a difference in conceptualising moral dimension as central parameter (e.g., Hunt–Vitell’s framework) versus additional predictor (e.g., the TPB) of intentions and behaviours raises some further theoretical questions. For example, if the measures of attitudes, subjective norms, and/or perceived behavioural control in the TPB already reflect some effects of the moral component, is it possible to dissociate the effects of moral vs. other dimensions in either of the constructs? Alternatively, if the effects of morality are already reflected in either or all of the TPB measures, does the additional measure of morality inflate the predictive capacity of the model?

The next question concerns our finding that morality did not translate into intentions-behaviours, despite being present in the attitudes. Here, obtained results pose the question of what prevented this from happening, and what other measures could have accounted for explaining the barrier between moral dimension and its translation into pro-environmental action? One possible explanation concerns the role of moral affect in pro-environmental vs. classical social dilemmas. Specifically, Markowitz and Shariff (2012) pointed out that climate change is a result of unintentional rather than intentional harm because no one wanted or intended for it to happen. According to the literature, unintentional harms are processed differently than intentional harms because the former fail to provoke a strong emotional reaction to moral transgressions (Ames and Fiske, 2013; Hesse et al., 2016). Therefore, it is possible that environmental harms do not generate strong intuitions, which in turn activate the motivation to analyse rights and wrongs (Decety et al., 2012). Indeed, the study by Zaikauskaite et al. (2020) has assessed the differences in the perception of morality in environmental vs. social cases and has found that effects of moral dimension were less strong in environmental scenarios, supporting the idea that moral intensity might be playing a role. If that would be the case, then it is possible that the events in the environmental scenarios were not processed in the same manner as they would have been processed in the classical moral scenarios depicting intentional moral transgressions (Cushman, 2008).

## Limitations and Future Directions

Admittedly, the present study is limited in some key respects. Foremost among these is the notion that we have measured self-reported rather than actual intentions and behaviours. Therefore, we cannot make a certain proposition that the findings from our study will correspond exactly to real-life scenarios [e.g., see Francis et al. (2016) for evidence on how the changes in the settings may impact the behaviour]. An experimental design that includes measuring actual rather than self-reported intentions and behaviours would help strengthen the methodological part of the experiment.

Second is the notion of the socially desirable response (Vilar et al., 2020). We aimed to reduce social desirability bias by (i) splitting the survey into two parts in order to tease apart the self-reporting of intentions vs. behaviours, (ii) attempting to reduce the error in the measurement of intention by incorporating two rather than one semantic differential scale, (iii) using 10 rather than 2 dilemmas in order to reduce context-induced measurement errors. Paired samples *t*-test results revealed significant differences between corresponding intention and behaviour items in all four conditions (see Supplementary Table 2), therefore it’s likely that the reduction of social desirability bias was satisfactory. However, it’s important to note the possibility that these efforts might have not been sufficient, and this is the reason why corresponding intention and behaviour items have loaded on the same rather than separate factors. The study using actual rather than self-reported measures is necessary to confirm the case.

Third, our research is limited by other minor factors, such as the sample composition (United States participants; Tata and Prasad, 2015), the number of dilemmas (Wark and Krebs, 2000; Christensen and Gomila, 2012), assumption of directional link between attitudes and behaviours (Kollmuss and Agyeman, 2002). We have not considered possible effects of sociodemographic characteristics, e.g., it could be that the sense of morality gets stronger with age and appropriate education (Buttel, 1979; Arcury, 1990). Importantly, we have not measured the effects of moral intensity (Morris and McDonald, 1995; Singhapakdi et al., 1999; May and Pauli, 2002). In fact, it would be interesting to see if varying moral intensity of the dilemma, e.g., altering the level of valence and arousal (Christensen et al., 2014; Zhang et al., 2017), would have a further influence on the relationship between morality and pro-environmental behaviours or perception of the sereneness of environmental transgression. Likewise, it would be interesting to investigate if the alternative mode of presentation, such as presenting environmental dilemmas using virtual reality rather than computer’s screen, would have any impact on the attitude-behaviour link (Francis et al., 2016, 2017).

## Conclusion

This study extends the research of the effects of morality in a pro-environmental domain. The results have demonstrated that the moral dimension failed to translate into pro-environmental intentions and behaviours, despite being integrated into the attitudes toward presented dilemmas. The findings suggest that (i) moral dimension had an effect in shaping ethical evaluations of presented dilemmas, and (ii) the “attitude-behaviour” gap occurred between attitudes and intentions rather than intentions and behaviours. Further investigation of what strengthens and/or overrides the effects of moral dimension would help understand the reasons why moral attitudes do not always translate into subsequent intentions and behaviours in the pro-environmental domain.

## Data Availability Statement

The datasets presented in this study can be found in online repositories. The names of the repository/repositories and accession number(s) can be found in the article/Supplementary Material.

## Ethics Statement

The studies involving human participants were reviewed and approved by University College London Ethics Committee. The patients/participants provided their written informed consent to participate in this study.

## Author Contributions

LZ: conceptualisation, data curation, investigation, project administration, writing – original draft preparation. LZ, CR, and LT: formal analyses. LZ and DT: funding acquisition. LZ, GB, and NH: methodology. DT and JD: supervision. LZ, LT, and JD: validation. LZ and CR: visualisation. LZ, GB, NH, CR, LT, DT, and JD: writing – review and editing. All authors contributed to the article and approved the submitted version.

## Conflict of Interest

LT was employed by Thomas International Limited. The remaining authors declare that the research was conducted in the absence of any commercial or financial relationships that could be construed as a potential conflict of interest.

## Publisher’s Note

All claims expressed in this article are solely those of the authors and do not necessarily represent those of their affiliated organizations, or those of the publisher, the editors and the reviewers. Any product that may be evaluated in this article, or claim that may be made by its manufacturer, is not guaranteed or endorsed by the publisher.

## Footnotes

1 Non-environmental attitudes are defined as the focus on non-environmental aspects of environmental problems such as laws rather than effects of the environmentally damaging actions on humans or on the environment itself (Tuncay et al., 2011, p. 169).

2 In this study, we use the term “teleology” in a non-standard way. Notably, our study conceptualises “teleology” as consequentialism (Mill, 1962; Mill and Bentham, 1987), whereas the original meaning of the term is much broader and refers to virtue ethics (Owens, 1968; Trianosky, 1990; Meyer, 1992).
