# Supplementary material for: Hunt–Vitell’s General Theory of Marketing Ethics Predicts “Attitude-Behaviour” Gap in Pro-environmental Domain
Source: Front Psychol. 2022 Mar 2;13:732661. doi: 10.3389/fpsyg.2022.732661 (PMC8924501; doi:10.3389/fpsyg.2022.732661)
Supplement: Supplementary file 1 [file Table_1.docx]

**Supplementary Materials**

*1. Experimental Protocol*

| **Research Design (2 x 2)**  **2-deontological behaviour (unethical, ethical) x 2-teleological consequences (positive, negative)** | | | |
| --- | --- | --- | --- |
| **Unethical behaviours** | | **Ethical behaviours** | |
| **Positive consequences** | **Negative consequences** | **Positive consequences** | **Negative consequences** |
| **Study version A** | **Study version B** | **Study version C** | **Study version D** |
| **a. Participant’s ID** | **a. Participant’s ID** | **a. Participant’s ID** | **a. Participant’s ID** |
| **1. Behaviours** | **1. Behaviours** | **1. Behaviours** | **1. Behaviours** |
| **2. Demographics** | **2. Demographics** | **2. Demographics** | **2. Demographics** |
| **b.** **Participant’s ID** | **b. Participant’s ID** | **b. Participant’s ID** | **b. Participant’s ID** |
| **3.1. – 3.2. Intention** | **3.1. – 3.2. Intention** | **3.1. – 3.2. Intention** | **3.1. – 3.2. Intention** |
| **4.1. Scenario: Random A** | **4.1. Scenario: Random B** | **4.1. Scenario: Random C** | **4.1. Scenario: Random D** |
| **5.1. Ethical Evaluation of 4.1.** | **5.1. Ethical Evaluation of 4.1.** | **5.1. Ethical Evaluation of 4.1.** | **5.1. Ethical Evaluation of 4.1.** |
| **c. Attention check item** | **c. Attention check item** | **c. Attention check item** | **c. Attention check item** |
| **4.2. Scenario: Random A** | **4.2. Scenario: Random B** | **4.2. Scenario: Random C** | **4.2. Scenario: Random D** |
| **5.2. Ethical Evaluation of 4.2.** | **5.2. Ethical Evaluation of 4.2.** | **5.2. Ethical Evaluation of 4.2.** | **5.2. Ethical Evaluation of 4.2.** |
| **d. Attention check item** | **d. Attention check item** | **d. Attention check item** | **d. Attention check item** |

*Note:*

*1. The experiment is launched in two Stages. Stage 1 contains items a, 1 & 2 and. Stage 2 contains items b, 3 – 5.2. Stages 1 and 2 conducted 5 weeks apart.*

*2. Items a, b, c, d do not contribute to the statistical analysis of the study.*

| **Study: The effectiveness of the General Theory of Marketing Ethics in predicting environmentally-(un)friendly behaviour** | |
| --- | --- |
| **a. Participant’s ID** | **Item** |
| *[Open answer]* | Please enter your MTurk ID |
| **1. Behaviours** | **Item** |
| *Likert: 1 (Never), 2 (Rarely), 3 (occasionally), 4 (Sometimes), 5 (Frequently), 6 (Usually), 7 (Every time)* | Please indicate the extent to which you are performing the following behaviors in your daily life:   1. Recycle newspapers, plastics, cans and glass 2. Compost kitchen waste 3. Turn off or unplug electronic devises when not need 4. Reduce air conditioning 5. Reduce driving, and walk, bike or use public transportation 6. Eat less meat and more vegetables 7. Buy local products or locally produced foods 8. Buy energy efficient appliances 9. Reduce using plastic bags, or use own bag when shopping 10. Bring own utensils when eating out |
| *Huang, H. (2016). Media use, environmental beliefs, self-efficacy, and pro-environmental behavior. Journal of Business Research, 69(6), 2206-2212* | |
| **2. Demographics** | **Item** |
| *1. [Open answer]* | Please type your current country of residence |
| *2. [Open answer]* | Please type your country of origin |
| *3. Multiple choice* | Please indicate your gender:   1. Male 2. Female 3. Prefer not to say |
| *4. Multiple choice* | Please indicate your age range:   1. 18-24 2. 25-34 3. 35-49 4. 50-64 5. 65 and over |
| *5. Multiple choice* | Please indicate your marital status:   1. Single (never married) 2. Married (no children) 3. Married (with children) 4. Domestic partnership 5. Divorced 6. Widowed 7. Separated |
| *6. Multiple choice* | Please indicate your level of education:   1. High school or less 2. Some college 3. Undergraduate 4. College graduate 5. Post collegiate 6. None of the above |
| *7. Multiple choice* | Please indicate your net monthly household income per annum:   1. Less than $9,999 2. $10,000 - $19,999 3. $20,000 - $29,999 4. $30,000 – 39,999 5. $40,000 - $49,999 6. $50,000 – 74,999 7. $75,000 or more |
| *8. Multiple choice* | Please indicate your employment status:   1. Full time 2. Part time 3. Self employed 4. Unemployed 5. Retired 6. Student 7. Other |
| **b. Participant’s ID** | **Item** |
| *[Open answer]* | Please enter your MTurk ID |
| **3.1. – 3.2. Intension** | **Item** |
| *7 point semantic differential scale with two anchor points (Unlikely – Likely)*  *7 point semantic differential scale with two anchor points (Undecided – Determined)* | Please rate the following statements.  I intend to:   1. Recycle newspapers, plastics, cans and glass 2. Compost kitchen waste 3. Turn off or unplugging electronic devises when not need 4. Reduce air conditioning 5. Reduce driving, and walk, bike or use public transportation 6. Eat less meat and more vegetables 7. Buy local products or locally produced foods 8. Buy energy efficient appliances 9. Reduce using plastic bags, or use own bag when shopping 10. Bring own utensils when eating out   *Response time measured.* |
| ***Technique taken from:*** *Kaiser, F. G., & Scheuthle, H. (2003). Two challenges to a moral extension of the theory of planned behavior: moral norms and just world beliefs in conservationism. Personality and individual differences, 35(5), 1033-1048*  *Kaiser, F. G. (2006). A moral extension of the theory of planned behavior: Norms and anticipated feelings of regret in conservationism. Personality and Individual Differences, 41(1), 71-81*  ***Scale taken from:*** *Huang, H. (2016). Media use, environmental beliefs, self-efficacy, and pro-environmental behavior. Journal of Business Research, 69(6), 2206-2212* | |
| **4.1. Scenario** | **Scenario** |
| *n/a* | Please read the following scenario:  [Insert scenario here]  *Scenario must correspond to the version of the experiment, e.g. version A = scenario A.*  *Presented randomly out of 10 scenarios.* |
| *Created by Laura & the Research Team. Inspiration from Hunt, S. D., & Vasquez-Parraga, A. Z. (1993). Organizational consequences, marketing ethics, and salesforce supervision. Journal of Marketing Research, 30(1), 78-90.* | |
| **5.1 Ethical Evaluation of 4.1.** | **Item** |
| *Likert: 1 (SD) – 7 (SA)* | Keeping the above scenario in mind, please state how much you disagree or agree with the following statements:  1. I consider x’s actions to be very ethical.  2. Most people would consider x’s actions to be very ethical.  *Presented on the same page with the scenario above.*  *Response time measured.* |
| *Vitell, S. J., Singhapakdi, A., & Thomas, J. (2001). Consumer ethics: an application and empirical testing of the Hunt-Vitell theory of ethics. Journal of Consumer marketing, 18(2), 153-178.* | |
| **c. Attention check item** | **Item** |
| [Open answer] | Please write 2-3 sentences summarising the key details of the scenario that has been presented in the previous question.  *Response time measured.* |
| **4.2. Scenario** | **Scenario** |
| *n/a* | Please read the following scenario:  [Insert scenario here]  *Scenario must correspond to the version of the experiment, e.g. version A = scenario A.*  *Presented randomly out of remaining 9 scenarios.* |
| *Created by Laura & the Research Team. Inspiration from Hunt, S. D., & Vasquez-Parraga, A. Z. (1993). Organizational consequences, marketing ethics, and salesforce supervision. Journal of Marketing Research, 30(1), 78-90.* | |
| **5.2. Ethical Evaluation of 4.2.** | **Item** |
| *Likert: 1 (SD) – 7 (SA)* | Keeping the above scenario in mind, please state how much you disagree or agree with the following statements:  1. I consider x’s actions to be very ethical.  2. Most people would consider x’s actions to be very ethical.  *Presented on the same page with the scenario above.*  *Response time measured.* |
| *Vitell, S. J., Singhapakdi, A., & Thomas, J. (2001). Consumer ethics: an application and empirical testing of the Hunt-Vitell theory of ethics. Journal of Consumer marketing, 18(2), 153-178.* | |
| **d. Manipulation check item** | **Item** |
| *[Open answer]* | Please write 2-3 sentences summarising the key details of the scenario that has been presented in the previous question.  *Response time measured.* |

*2. Scenarios*

**Scenario 1: Recycling newspapers, plastics, cans and glass**

| Scenario 1 | **Deontologically unethical behaviour / Code 0** | **Deontologically ethical behaviour / Code 1** |
| --- | --- | --- |
| **Teleological:**  **Positive consequences of main character’s behaviour to the environment / Code 1** | **1A. Scene:** John and his teammates are in charge of the props for the school’s theatre production. Some of the materials used to make the props were glass, cans newspapers and plastics. John has decided to not keep any of the props after they were used due to lack of storage, but there are only 10 minutes left to clear the props before the premises closes. | **1C. Scene:** John and his teammates are in charge of the props for the school’s theatre production. Some of the materials used to make the props were glass, cans newspapers and plastics. John has decided to not keep any of the props after they were used due to lack of storage, but there are only 10 minutes left to clear the props before the premises closes. |
|  | **Environmental case:** John knows that not recycling will lead to factories constantly producing new materials, which requires depletion of natural resources. Recycling helps save natural resources, energy, and reduce the carbon emission pollution that would come from producing new materials every time. | **Environmental case:** John knows that not recycling will lead to factories constantly producing new materials, which requires depletion of natural resources. Recycling helps save natural resources, energy, and reduce the carbon emission pollution that would come from producing new materials every time. |
|  | **Deont. Behaviour** unethical: Given the situation, John decides to throw away the props since they are running out of time. | **Deont. Behaviour** ethical: Given the situation, John decides to tell his teammates to bring the props home due to shortage of time, and plans to meet up the next day to sort out the props based on recycling category. |
|  | **Circumstances.** However, his teammates decide to bring the props home so they could recycle them the next day. | **Circumstances**. n/a |
|  | **Teleol. Conseq.** (positive for the environment): The teammates’ decision means that the props will be recycled and will not end up in the landfill. | **Teleol. Conseq**. (positive for the environment): John’s decision means that the props will be recycled and will not end up in the landfill. |
| **Teleological:**  **Negative consequences of main character’s behaviour to the environment / Code 0** | **1B. Scene:** John and his teammates are in charge of the props for the school’s theatre production. Some of the materials used to make the props were glass, cans newspapers and plastics. John has decided to not keep any of the props after they were used due to lack of storage, but there are only 10 minutes left to clear the props before the premises closes. | **1D. Scene:** John and his teammates are in charge of the props for the school’s theatre production. Some of the materials used to make the props were glass, cans newspapers and plastics. John has decided to not keep any of the props after they were used due to lack of storage, but there are only 10 minutes left to clear the props before the premises closes. |
|  | **Environmental case:** **Environmental case:** John knows that not recycling will lead to factories constantly producing new materials, which requires depletion of natural resources. Recycling helps save natural resources, energy, and reduce the carbon emission pollution that would come from producing new materials every time. | **Environmental case:** **Environmental case:** John knows that not recycling will lead to factories constantly producing new materials, which requires depletion of natural resources. Recycling helps save natural resources, energy, and reduce the carbon emission pollution that would come from producing new materials every time. |
|  | **Deont. Behaviour** unethical: Given the situation, John decides to throw away the props since they are running out of time. | **Deont. Behaviour** ethical: Given the situation, John decides to tell his teammates to bring the props home due to shortage of time, and plans to meet up the next day to sort out the props based on recycling category. |
|  | **Circumstances**. n/a | **Circumstances**. However, his teammates think it’s too much work and simply throw the props away without recycling. |
|  | **Teleol. Conseq.** (negative for the environment): John’s decision means that the props will not be recycled and will therefore end up in a landfill. | **Teleol. Conseq.** (negative for the environment): The teammates’ decision means that the props will not be recycled and will therefore end up in a landfill. |

**Scenario 2: Composting kitchen waste**

| Scenario 2 | **Deontologically unethical behaviour / Code 0** | **Deontologically ethical behaviour / Code 1** |
| --- | --- | --- |
| **Teleological:**  **Positive consequences of main character’s behaviour to the environment / Code 1** | **2A.** **Scene:** Stella has just prepared a big meal for her household and has a lot of leftover food and kitchen waste. Stella has a kitchen compost bin in her garden, but has recently been receiving complaints from her neighbours about the odour of the bin. | **2C.** **Scene:** Stella has just prepared a big meal for her household and has a lot of leftover food and kitchen waste. Stella has a kitchen compost bin in her garden, but has recently been receiving complaints from her neighbours about the odour of the bin. |
|  | **Environmental case:** Stella normally composts her kitchen waste to keep food waste out of landfills where it gets decomposed anaerobically, which is a major cause of greenhouse gas emissions. | **Environmental case:** Stella normally composts her kitchen waste to keep food waste out of landfills where it gets decomposed anaerobically, which is a major cause of greenhouse gas emissions. |
|  | **Deont. Behaviour** unethical: To save getting complaints from her neighbours again, Stella decides to stop using the compost bin. | **Deont. Behaviour** ethical: Stella decides that she doesn’t mind being told off by her neighbours again and puts today’s kitchen waste into the compost bin. |
|  | **Circumstances**. However, her flatmate says he will take care of kitchen waste and secretly puts everything into the compost bin. | **Circumstances**. n/a |
|  | **Teleol. Conseq.** (positive for the environment): The flatmate’s decision means that they will receive complaints from neighbours again, but will not add to the greenhouse gas problem. | **Teleol. Conseq.** (positive for the environment): Stella’s decision means that they will receive complaints from neighbours again, but will not add to the greenhouse gas problem. |
| **Teleological:**  **Negative consequences of main character’s behaviour to the environment / Code 0** | **2B.** **Scene:** Stella has just prepared a big meal for her household and has a lot of leftover food and kitchen waste. Stella has a kitchen compost bin in her garden, but has recently been receiving complaints from her neighbours about the odour of the bin. | **2D.** **Scene:** Stella has just prepared a big meal for her household and has a lot of leftover food and kitchen waste. Stella has a kitchen compost bin in her garden, but has recently been receiving complaints from her neighbours about the odour of the bin. |
|  | **Environmental case:** Stella normally composts her kitchen waste to keep food waste out of landfills where it gets decomposed anaerobically, which is a major cause of greenhouse gas emissions. | **Environmental case:** Stella normally composts her kitchen waste to keep food waste out of landfills where it gets decomposed anaerobically, which is a major cause of greenhouse gas emissions. |
|  | **Deont. Behaviour** unethical: To save getting complaints from her neighbours again, Stella decides to stop using the compost bin. | **Deont. Behaviour** ethical: Stella decides that she doesn’t mind being told off by her neighbours again. |
|  | **Circumstances**. n/a | **Circumstances**. However, her flatmate reminds how annoying it is to ruin relationships with neighbours, and to constantly hear complaints. Therefore, the flatmate simply throws away all kitchen waste without composting it. |
|  | **Teleol. Conseq.** (negative for the environment): Stella’s decision to stop composting means that she will add to the greenhouse gas problem. | **Teleol. Conseq.** (negative for the environment): The flatmate’s decision not to compost kitchen waste means that they have added to the greenhouse gas problem. |

**Scenario 3: Turn off or unplug electronic devices when not in need**

| Scenario 3 | **Deontologically unethical behaviour / Code 0** | **Deontologically ethical behaviour / Code 1** |
| --- | --- | --- |
| **Teleological:**  **Positive consequences of main character’s behaviour to the environment / Code 1** | **3A. Scene:** Tim and his friend are going to a concert and have just left the house. The next bus will be arriving in 15 minutes and they have already been walking for 5 minutes. Suddenly, Tim remembers that he has forgotten to switch off the lights at home. | **3C. Scene:** Tim and his friend are going to a concert and have just left the house. The next bus will be arriving in 15 minutes and they have already been walking for 5 minutes. Suddenly, Tim remembers that he has forgotten to switch off the lights at home. |
|  | **Environmental case:** Tim’s neighbourhood has agreed to support environment friendly practices by saving electricity, especially switching off electrical appliances when not in use. | **Environmental case:** Tim’s neighbourhood has agreed to support environment friendly practices by saving electricity, especially switching off electrical appliances when not in use. |
|  | **Deont. Behaviour** unethical: Tim decides not to tell his friend because returning back home would mean that they would miss the next bus. This will result in both of them being late to the concert. | **Deont. Behaviour** ethical: Tim decides to tell his friend, although returning back home would mean that they would miss the next bus. This will result in both of them being late to the concert. Tim’s friend follows him back home to double check if other electrical appliances have been switched off. |
|  | **Circumstances**. However, Tim’s friend has just asked if they have switched the lights off. Friend insists on returning back home and switch them off. | **Circumstances**. n/a |
|  | **Teleol. Conseq.** (positive for the environment): The friend’s decision means that he and Tim will be late for the concert, but they will support environmentally friendly lifestyle by saving electricity at home. | **Teleol. Conseq.** (positive for the environment): Tim’s decision means Tim and his friend will be late for the concert, but they will support environmentally friendly lifestyle by saving electricity at home. |
| **Teleological:**  **Negative consequences of main character’s behaviour to the environment / Code 0** | **3B.** **Scene:** Tim and his friend are going to a concert and have just left the house. The next bus will be arriving in 15 minutes and they have already been walking for 5 minutes. Suddenly, Tim remembers that he has forgotten to switch off the lights at home. | **3D. Scene:** Tim and his friend are going to a concert and have just left the house. The next bus will be arriving in 15 minutes and they have already been walking for 5 minutes. Suddenly, Tim remembers that he has forgotten to switch off the lights at home. |
|  | **Environmental case:** Tim’s neighbourhood has agreed to support environment friendly practices by saving electricity, especially switching off electrical appliances when not in use. | **Environmental case:** Tim’s neighbourhood has agreed to support environment friendly practices by saving electricity, especially switching off electrical appliances when not in use. |
|  | **Deont. Behaviour** unethical: Tim decides not to tell his friend because returning back home would mean that they would miss the next bus. This would result in both of them being late to the concert. | **Deont. Behaviour** ethical: Tim decides to tell his friend, although returning back home would mean that they would miss the next bus. This will result in both of them being late to the concert. |
|  | **Circumstances**. n/a | **Circumstances**. However, Tim’s friend insists on continuing the trip to the concert without returning home. |
|  | **Teleol. Conseq.** (negative for the environment): Tim’s decision means that he and his friend will be on time for the concert, but the neighbourhood’s effort to reduce electricity consumption will be hindered due to the amount of electricity wasted. | **Teleol. Conseq.** (negative for the environment): The friend’s decision means Tim and his friend will be on time for the concert, but the neighbourhood’s effort to reduce electricity consumption will be hindered due to the amount of electricity wasted. |

**Scenario 4: Reducing air conditioning**

| Scenario 4 | **Deontologically unethical behaviour / Code 0** | **Deontologically ethical behaviour / Code 1** |
| --- | --- | --- |
| **Teleological:**  **Positive consequences of main character’s behaviour to the environment / Code 1** | **4A**. **Scene:** Anna’s friend is staying over at hers for a sleepover. It is the middle of July, and it is really warm. When going to sleep, Anna’s friend asks if she can turn on the air conditioning (AC) in her room and keep it on until the morning. She agrees to turn it on, as she is feeling quite hot herself, but is unsure about whether she should keep it on until the morning. Turning it off before going to sleep would also keep the room cool for a while. | **4C**. **Scene:** Anna’s friend is staying over at hers for a sleepover. It is the middle of July, and it is really warm. When going to sleep, Anna’s friend asks if she can turn on the air conditioning (AC) in her room and keep it on until the morning. She agrees to turn it on, as she is feeling quite hot herself, but is unsure about whether she should keep it on until the morning. Turning it off before going to sleep would also keep the room cool for a while. |
|  | **Environmental case:** Anna knows that air conditioners pose extra demand for power and electricity, which not only inflates the electricity bill but also leads to more pollution, as the generation of electricity relies mostly on the burning of coal. Also, there is a release of poisonous gases into the environment (such as chlorofluorocarbons and hydro- chlorofluorocarbons), which are a part of the greenhouse gases, contributing to the depletion of the ozone layer and global warming. | **Environmental case:** Anna knows that air conditioners pose extra demand for power and electricity, which not only inflates the electricity bill but also leads to more pollution, as the generation of electricity relies mostly on the burning of coal. Also, there is a release of poisonous gases into the environment (such as chlorofluorocarbons and hydro- chlorofluorocarbons), which are a part of the greenhouse gases, contributing to the depletion of the ozone layer and global warming. |
|  | **Deont. Behaviour** unethical: However, Anna decides it’s better to keep the AC on throughout the night; her friend’s happiness quality of sleep is more important. | **Deont. Behaviour** ethical: However, Anna decides it is better to turn the AC off. |
|  | **Circumstances**. Eventually, Anna wakes up the next morning to see that the AC is off. Her friend said she felt cold in the middle of the night and woke up to switch it off. | **Circumstances**. n/a |
|  | **Teleol. Conseq.** (positive for the environment): The friend’s decision means they have not contributed to the large energy consumption problem and the resulting pollution. | **Teleol. Conseq.** (positive for the environment): Anna’s decision means they have not contributed to the large energy consumption problem and the resulting pollution. |
| **Teleological:**  **Negative consequences of main character’s behaviour to the environment / Code 0** | **4B**. **Scene:** Anna’s friend is staying over at hers for a sleepover. It is the middle of July, and it is really warm. When going to sleep, Anna’s friend asks if she can turn on the air conditioning (AC) in her room and keep it on until the morning. She agrees to turn it on, as she is feeling quite hot herself, but is unsure about whether she should keep it on until the morning. Turning it off before going to sleep would also keep the room cool for a while. | **4D**. **Scene:** Anna’s friend is staying over at hers for a sleepover. It is the middle of July, and it is really warm. When going to sleep, Anna’s friend asks if she can turn on the air conditioning (AC) in her room and keep it on until the morning. She agrees to turn it on, as she is feeling quite hot herself, but is unsure about whether she should keep it on until the morning. Turning it off before going to sleep would also keep the room cool for a while. |
|  | **Environmental case:** Anna knows that air conditioners pose extra demand for power and electricity, which not only inflates the electricity bill but also leads to more pollution, as the generation of electricity relies mostly on the burning of coal. Also, there is a release of poisonous gases into the environment (such as chlorofluorocarbons and hydro- chlorofluorocarbons), which are a part of the greenhouse gases, contributing to the depletion of the ozone layer and global warming. | **Environmental case:** Anna knows that air conditioners pose extra demand for power and electricity, which not only inflates the electricity bill but also leads to more pollution, as the generation of electricity relies mostly on the burning of coal. Also, there is a release of poisonous gases into the environment (such as chlorofluorocarbons and hydro- chlorofluorocarbons), which are a part of the greenhouse gases, contributing to the depletion of the ozone layer and global warming. |
|  | **Deont. Behaviour** unethical: However, Anna decides it’s better to keep the AC on throughout the night; her friend’s happiness quality of sleep is more important. | **Deont. Behaviour** ethical: However, Anna decides it is better to turn the AC off. |
|  | **Circumstances.** n/a | **Circumstances.** Anna wakes up the next morning and sees the AC working on full blast. Her friend says she got hot during the night and turned the AC on. |
|  | **Teleol. Conseq.** (negative for the environment): Anna’s decision means that the AC was working throughout the night in full blast, consuming energy for 10 additional hours than if it had been switched off. This means it has contributed to the large energy consumption problem and the resulting pollution. | **Teleol. Conseq.** (negative for the environment): The friend’s decision means that the AC was working throughout the night in full blast, consuming energy for 10 additional hours than if it had been switched off. This means it has contributed to the large energy consumption problem and the resulting pollution. |

**Scenario 5: Reducing driving, and walk, bike or use public transportation**

| Scenario 5 | **Deontologically unethical behaviour / Code 0** | **Deontologically ethical behaviour / Code 1** |
| --- | --- | --- |
| **Teleological:**  **Positive consequences of main character’s behaviour to the environment / Code 1** | **5A. Scene:** It is 6 PM, the end of a very busy working day, and is finally time to go home. Victor is contemplating whether to walk or to call a taxi. Victor is in a rush to go home, as he has promised to attend a friend’s birthday dinner at 7 PM. Victor is tired, sleep deprived, hungry, and is carrying several bags, all of which are heavy. He lives 20 minutes away by walk and 15 minutes away by taxi. | **5C. Scene:** It is 6 PM, the end of a very busy working day, and is finally time to go home. Victor is contemplating whether to walk or to call a taxi. Victor is in a rush to go home, as he has promised to attend a friend’s birthday dinner at 7 PM. Victor is tired, sleep deprived, hungry, and is carrying several bags, all of which are heavy. He lives 20 minutes away by walk and 15 minutes away by taxi. |
|  | **Environmental case:** Going home by a taxi will be faster and more comfortable, however for the same journey he could walk, Victor’s choice will result in burning petrol, and releasing greenhouse gases which contribute to the depletion of the ozone layer and global warming. | **Environmental case:** Going home by a taxi will be faster and more comfortable, however for the same journey he could walk, Victor’s choice will result in burning petrol, and releasing greenhouse gases which contribute to the depletion of the ozone layer and global warming. |
|  | **Deont. Behaviour** unethical: Victor decides he is too tired to bare the 20 minute walk home, so he calls a taxi. | **Deont. Behaviour** ethical: However, Victor decides it is not worth it to call a taxi and burn petrol only to get home 5 minutes faster and in a more comfortable manner. He walks home instead. |
|  | **Circumstances**. However, the taxis are quite busy, and the soonest time a taxi would be available to pick him up is in 20 minutes, which would mean he will reach home in 35 minutes. Luckily, his colleague who drives an electric car passes by and offers him a lift home. | **Circumstances**. n/a |
|  | **Teleol. Conseq.** (positive for the environment): The colleague’s decision to help Victor out means that his journey is carbon-free and will not contribute to the burning of petrol and releasing greenhouse gasses. | **Teleol. Conseq.** (positive for the environment): Victor’s decision means that his journey is carbon-free and will not contribute to the burning of petrol and releasing greenhouse gasses. |
| **Teleological:**  **Negative consequences of main character’s behaviour to the environment / Code 0** | **5B. Scene:** It is 6 PM, the end of a very busy working day, and is finally time to go home. Victor is contemplating whether to walk or to call a taxi. Victor is in a rush to go home, as he has promised to attend a friend’s birthday dinner at 7 PM. Victor is tired, sleep deprived, hungry, and is carrying several bags, all of which are heavy. He lives 20 minutes away by walk and 15 minutes away by taxi. | **5D. Scene:** It is 6 PM, the end of a very busy working day, and is finally time to go home. Victor is contemplating whether to walk or to call a taxi. Victor is in a rush to go home, as he has promised to attend a friend’s birthday dinner at 7 PM. Victor is tired, sleep deprived, hungry, and is carrying several bags, all of which are heavy. He lives 20 minutes away by walk and 15 minutes away by taxi. |
|  | **Environmental case:** Going home by a taxi will be faster and more comfortable, however for the same journey he could walk, Victor’s choice will result in burning petrol, and releasing greenhouse gases which contribute to the depletion of the ozone layer and global warming. | **Environmental case:** Going home by a taxi will be faster and more comfortable, however for the same journey he could walk, Victor’s choice will result in burning petrol, and releasing greenhouse gases which contribute to the depletion of the ozone layer and global warming. |
|  | **Deont. Behaviour** unethical: Victor decides he is too tired to bare the 20 minute walk home, so he calls a taxi. | **Deont. Behaviour** ethical: However, Victor decides it is not worth it to call a taxi and burn petrol to only get home 5 minutes sooner and in a more comfortable manner. He walks home instead. |
|  | **Circumstances**. n/a | **Circumstances**. In the first 5 minutes of his walk, Victor’s backpack strap breaks. It’s hard for him to can’t carry it on one shoulder, as his bag is too heavy and he has other bags to carry. At this point, a colleague sees Victor struggling and calls a taxi. |
|  | **Teleol. Conseq.** (negative for the environment): Victor’s decision means that Victor’s journey is not carbon-free and will contribute to the burning of petrol and releasing greenhouse gasses. | **Teleol. Conseq.** (negative for the environment): The colleague’s decision means that Victor’s journey is not carbon-free and will contribute to the burning of petrol and releasing greenhouse gasses. |

**Scenario 6: Eat less meat and more vegetables**

| Scenario 6 | **Deontologically unethical behaviour / Code 0** | **Deontologically ethical behaviour / Code 1** |
| --- | --- | --- |
| **Teleological:**  **Positive consequences of main character’s behaviour to the environment / Code 1** | **6A.** **Scene:** It is Monday and Ines has met her friend at a local restaurant for a dinner. Ines selected the restaurant as it is well-known for its tasty vegetarian options, but upon arrival she sees her favourite dish on the menu, beef bolognaise. The waiter arrives at a table and asks to take the order. | **6C.** **Scene:** It is Monday and Ines has met her friend at a local restaurant for a dinner. Ines selected the restaurant as it is well-known for its tasty vegetarian options, but upon arrival she sees her favourite dish on the menu, beef bolognaise. The waiter arrives at a table and asks to take the order. |
|  | **Environmental case:** Ines normally takes part in ‘meat-free Monday’ to reduce the amount of meat and fish people consume and lessen the greenhouse gas emissions that occur from the farming of livestock. | **Environmental case:** Ines normally takes part in ‘meat-free Monday’ to reduce the amount of meat and fish people consume and lessen the greenhouse gas emissions that occur from the farming of livestock. |
|  | **Deont. Behaviour unethical:** Despite the fact it is Monday, Ines decides to order the beef bolognaise. | **Deont. Behaviour** ethical: Even though beef bolognaise is her favourite dish, Ines decides to stick to meat-free Monday and order a vegetarian option. |
|  | **Circumstances**. However, after ordering, the waiter returns to a table to advise that the restaurant has run out of the beef bolognaise and offers her a vegan option. | **Circumstances**. n/a |
|  | **Teleol. Conseq. (positive for environment):** Restaurant’s situation means that Ines takes part in ‘meat-free Monday’ and helps support the reduction of greenhouse gases caused by the farming livestock. | **Teleol. Conseq. (positive for environment):** Ines decision means she takes part in ‘meat-free Monday’ and helps support the reduction of greenhouse gases caused by the farming livestock. |
| **Teleological:**  **Negative consequences of main character’s behaviour to the environment / Code 0** | **6B.** **Scene:** It is Monday and Ines has met her friend at a local restaurant for a dinner. Ines selected the restaurant as it is well-known for its tasty vegetarian options, but upon arrival she sees her favourite dish on the menu, beef bolognaise. The waiter arrives at a table and asks to take the order. | **6D.** **Scene:** It is Monday and Ines has met her friend at a local restaurant for a dinner. Ines selected the restaurant as it is well-known for its tasty vegetarian options, but upon arrival she sees her favourite dish on the menu, beef bolognaise. The waiter arrives at a table and asks to take the order. |
|  | **Environmental case:** Ines normally takes part in ‘meat-free Monday’ to reduce the amount of meat and fish people consume and lessen the greenhouse gas emissions that occur from the farming of livestock. | **Environmental case:** Ines normally takes part in ‘meat-free Monday’ to reduce the amount of meat and fish people consume and lessen the greenhouse gas emissions that occur from the farming of livestock. |
|  | **Deont. Behaviour unethical:** Despite the fact it is Monday, Ines decides to order the beef bolognaise. | **Deont. Behaviour ethical:** Even though beef bolognaise is her favourite dish, Ines decides to stick to ‘meat-free Monday’ and order a vegetarian option. |
|  | **Circumstances**. n/a | **Circumstances**. However, her friend suggests they should share the charcuterie (meat) board. |
|  | **Teleol. Conseq. (negative for environment):** Ines’ decision means that she does not take part in ‘meat-free Monday’ and will not help support the reduction of greenhouse gases caused by the farming livestock. | **Teleol. Conseq. (negative for environment):** The friend’s decision means that Ines does not take part in ‘meat-free Monday’ and will not help support the reduction of greenhouse gases caused by the farming livestock. |

**Scenario 7: Buy local products or locally produced goods**

| Scenario 7 | **Deontologically unethical behaviour / Code 0** | **Deontologically ethical behaviour / Code 1** |
| --- | --- | --- |
| **Teleological:**  **Positive consequences of main character’s behaviour to the environment / Code 1** | **7A. Scene:** Dimitrios and his friend decided to cook dinner together this evening. They both went to a favourite shop nearby and his friend suggested to buy pasta, which is cheap and tasty. | **7C. Scene:** Dimitrios and his friend decided to cook dinner together this evening. They both went to a favourite shop nearby and his friend suggested to buy pasta, which is cheap and tasty. |
|  | **Environmental case:** Dimitrios knows that this particular pasta tastes good, but he also knows that this company uses unsustainable practices in its production. There is a choice of locally produced pasta, however it is twice as much expensive. | **Environmental case:** Dimitrios knows that this particular pasta tastes good, but he also knows that this company uses unsustainable practices in its production. There is a choice of locally produced pasta, however it is twice as much expensive. |
|  | **Deont. Behaviour unethical:** Dimitrios decides not to point this out to his friend and agrees with the cheap pasta option. | **Deont. Behaviour ethical:** Dimitrios decides to point this out to his friend and suggests to buying the locally produced pasta, despite it being more expensive. |
|  | **Circumstances**. However, his friend notices the option of locally produced pasta and suggests purchasing that, despite it being more expensive. | **Circumstances**. n/a |
|  | **Teleol. Conseq. (positive for environment):** Friend’s decision means that the cost will be higher, but the final outcome will support the production of locally produced goods which help save the environment. | **Teleol. Conseq. (positive for environment):** Dimitrios’ decision means that the cost will be higher, but the final outcome will support the production of locally produced goods which help save the environment. |
| **Teleological:**  **Negative consequences of main character’s behaviour to the environment / Code 0** | **7B. Scene:** Dimitrios and his friend decided to cook dinner together this evening. They both went to a favourite shop nearby and his friend suggested to buy pasta, which is cheap and tasty. | **7D. Scene:** Dimitrios and his friend decided to cook dinner together this evening. They both went to a favourite shop nearby and his friend suggested to buy pasta, which is cheap and tasty. |
|  | **Environmental case:** Dimitrios knows that this particular pasta tastes good, but he also knows that this company uses unsustainable practices in its production. There is a choice of locally produced pasta, however it is twice as much expensive. | **Environmental case:** Dimitrios knows that this particular pasta tastes good, but he also knows that this company uses unsustainable practices in its production. There is a choice of locally produced pasta, however it is twice as much expensive. |
|  | **Deont. Behaviour unethical:** Dimitrios decides not to point this out to his friend and agrees with the cheap pasta option. | **Deont. Behaviour ethical:** Dimitrios decides to point this out to his friend and suggests buying the locally produced pasta, despite it being more expensive. |
|  | **Circumstances**. n/a | **Circumstances**. However, his friend prefers to save money, therefore decides to buy the cheap pasta. |
|  | **Teleol. Conseq. (negative for environment):** Dimitrios’ decision means that the pasta will be cheaper, but the final outcome will not support the production of locally produced goods which help save the environment. | **Teleol. Conseq. (negative for environment):** The friend’s decision means that the pasta will be cheaper, but the final outcome will not support the production of locally produced goods which help save the environment. |

**Scenario 8: Buy energy efficient appliances**

| Scenario 8 | **Deontologically unethical behaviour / Code 0** | **Deontologically ethical behaviour / Code 1** |
| --- | --- | --- |
| **Teleological:**  **Positive consequences of main character’s behaviour to the environment / Code 1** | **8A. Scene:** Laura’s old kettle has broken so she decides to buy a new one. She goes to a shop and browses the selection. The shop sells a range of kettles, some of which are energy efficient. But none of the energy efficient kettles will match her kitchen décor. | **8C. Scene:** Laura’s old kettle has broken so she decides to buy a new one. She goes to a shop and browses the selection. The shop sells a range of kettles, some of which are energy efficient. But none of the energy efficient kettles will match her kitchen décor. |
|  | **Environmental case:** Laura knows that energy efficient appliances reduce fossil fuel use and greenhouse gas emissions, which helps protect the environment. | **Environmental case:** Laura knows that energy efficient appliances reduce fossil fuel use and greenhouse gas emissions, which helps protect the environment. |
|  | **Deont. Behaviour unethical:** However, Laura wants a kettle that is in keeping with her kitchen décor and therefore buys a non-energy efficient kettle. | **Deont. Behaviour ethical:** However, Laura decides that it does not matter if the kettle does not match her kitchen décor and buys an energy efficient kettle. |
|  | **Circumstances**. When she reaches the till point, the cashier informs that they have a very similar style kettle that is energy efficient which she hadn’t seen. | **Circumstances**. n/a |
|  | **Teleol. Conseq. (positive for environment):** Cashier’s decision results in Laura buying the energy efficient kettle and consequently helps protect the environment. | **Teleol. Conseq. (positive for environment):** Laura’s decision means that she buys the energy efficient kettle and consequently helps protect the environment. |
| **Teleological:**  **Negative consequences of main character’s behaviour to the environment / Code 0** | **8B. Scene:** Laura’s old kettle has broken so she decides to buy a new one. She goes to a shop and browses the selection. The shop sells a range of kettles, some of which are energy efficient. But none of the energy efficient kettles will match her kitchen décor. | **8D. Scene:** Laura’s old kettle has broken so she decides to buy a new one. She goes to a shop and browses the selection. The shop sells a range of kettles, some of which are energy efficient. But none of the energy efficient kettles will match her kitchen décor. |
|  | **Environmental case:** Laura knows that energy efficient appliances reduce fossil fuel use and greenhouse gas emissions, which helps protect the environment. | **Environmental case:** Laura knows that energy efficient appliances reduce fossil fuel use and greenhouse gas emissions, which helps protect the environment. |
|  | **Deont. Behaviour unethical:** However, Laura wants a kettle that is in keeping with her kitchen décor and therefore buys a non-energy efficient kettle. | **Deont. Behaviour ethical:** However, Laura decides that it does not matter if the kettle does not match her kitchen décor and buys an energy efficient kettle. |
|  | **Circumstances**. n/a | **Circumstances**. When she gets home, Laura notices that the kettle is faulty and does not turn on. She calls the company, but they replace it with the one that is not energy efficient. |
|  | **Teleol. Conseq. (negative for environment):** Laura’s decision means that the new kettle will use more energy and will not help protect the environment. | **Teleol. Conseq. (negative for environment):** Company’s decision means that the new kettle will use more energy and will not help protect the environment. |

**Scenario 9: Reduce using plastic bags, or use own bag when shopping**

| Scenario 9 | **Deontologically unethical behaviour / Code 0** | **Deontologically ethical behaviour / Code 1** |
| --- | --- | --- |
| **Teleological:**  **Positive consequences of main character’s behaviour to the environment / Code 1** | **9A. Scene:** Peter and his flatmate have just completed their weekly food shop at the supermarket and are packing their purchases in the reusable bags that they brought with them. As they are packing, Peter notices that the handle on one of the bags is broken. They don’t have far to walk home and, although awkward, Peter could carry the bag with only one handle and fix it at home. | **9C. Scene:** Peter and his flatmate have just completed their weekly food shop at the supermarket and are packing their purchases in the reusable bags that they brought with them. As they are packing, Peter notices that the handle on one of the bags is broken. They don’t have far to walk home and, although awkward, Peter could carry the bag with only one handle and fix it at home. |
|  | **Environmental case:** Peter normally uses reusable bags as it can take over 1000 years for a plastic bag to decompose, and this has severe effects on the environment. | **Environmental case:** Peter normally uses reusable bags as it can take over 1000 years for a plastic bag to decompose, and this has severe effects on the environment. |
|  | **Deont. Behaviour unethical:** Eventually, Peter decides that it’s too much hassle to carry the reusable bag with one handle and asks the cashier for a 5p plastic bag. | **Deont. Behaviour ethical:** Peter decides that he doesn’t mind that it will be more difficult to carry the bag home, and so doesn’t buy a 5p bag. |
|  | **Circumstances**. However, his flatmate says that he will carry the bag instead, so Peter doesn’t need to buy a plastic 5p bag. | **Circumstances**. n/a |
|  | **Teleol. Conseq. (positive for environment):** The flatmate’s decision means that he may struggle to carry the bag home, but the final outcome will not result in adding to the plastic problem by buying an unnecessary plastic bag. | **Teleol. Conseq. (positive for environment):** Peter’s decision means that he may struggle to carry the bag home, but the final outcome will not result in adding to the plastic problem by buying an unnecessary plastic bag. |
| **Teleological:**  **Negative consequences of main character’s behaviour to the environment / Code 0** | **9B. Scene:** Peter and his flatmate have just completed their weekly food shop at the supermarket and are packing their purchases in the reusable bags that they brought with them. As they are packing, Peter notices that the handle on one of the bags is broken. They don’t have far to walk home and, although awkward, Peter could carry the bag with only one handle and fix it at home. | **9D. Scene:** Peter and his flatmate have just completed their weekly food shop at the supermarket and are packing their purchases in the reusable bags that they brought with them. As they are packing, Peter notices that the handle on one of the bags is broken. They don’t have far to walk home and, although awkward, Peter could carry the bag with only one handle and fix it at home. |
|  | **Environmental case:** Peter normally uses reusable bags as it can take over 1000 years for a plastic bag to decompose, and this has severe effects on the environment. | **Environmental case:** Peter normally uses reusable bags as it can take over 1000 years for a plastic bag to decompose, and this has severe effects on the environment. |
|  | **Deont. Behaviour unethical:** Eventually, Peter decides that it’s too much hassle to carry the reusable bag with one handle and asks the cashier for a 5p plastic bag. | **Deont. Behaviour ethical:** Peter decides that he doesn’t mind that it will be more difficult to carry the bag home, and so doesn’t buy a plastic 5p bag. |
|  | **Circumstances**. n/a | **Circumstances**. However, the cashier notices that his bag is broken and gives him a free plastic bag at the checkout. |
|  | **Teleol. Conseq. (negative for environment):** Peter’s decision means that his action will result in adding to the plastic problem by buying an unnecessary plastic bag. | **Teleol. Conseq. (negative for environment):** The cashier’s decision bag means that this action will result in adding to the plastic problem by using an unnecessary plastic bag. |

**Scenario 10: Bring own utensils when eating out**

| Scenario 10 | **Deontologically unethical behaviour / Code 0** | **Deontologically ethical behaviour / Code 1** |
| --- | --- | --- |
| **Teleological:**  **Positive consequences of main character’s behaviour to the environment / Code 1** | **10A. Scene:** It’s lunchtime so Cindy leaves the office and heads to a café with her colleagues. The café provides throwaway plastic cutlery (knives, forks etc.) to use, but Cindy normally brings her own metal, washable cutlery instead. Once she places her order, Cindy realises that she has left her cutlery at the office. The café is only a few minutes’ walk from the office, but it has started raining outside and she hasn’t brought an umbrella. | **10C. Scene:** It’s lunchtime so Cindy leaves the office and heads to a café with her colleagues. The café provides throwaway plastic cutlery (knives, forks etc.) to use, but Cindy normally brings her own metal, washable cutlery instead. Once she places her order, Cindy realises that she has left her cutlery at the office. The café is only a few minutes’ walk from the office, but it has started raining outside and she hasn’t brought an umbrella. |
|  | **Environmental case:** Cindy knows that the plastic cutlery goes to landfill sites and pollutes the environment, which is why she and her colleagues normally bring their own. | **Environmental case:** Cindy knows that the plastic cutlery goes to landfill sites and pollutes the environment, which is why she and her colleagues normally bring their own. |
|  | **Deont. Behaviour unethical:** Cindy decides that she doesn’t want to get wet and picks up some plastic cutlery. | **Deont. Behaviour ethical:** Cindy decides it doesn’t matter if she will get wet and heads back to the office to pick up her cutlery. |
|  | **Circumstances**. However, her colleague sees her and says Cindy can use her cutlery, as she has ordered a sandwich and does not need to use her own. | **Circumstances**. n/a |
|  | **Teleol. Conseq. (positive for environment):** The colleague’s decision means that there won’t be an unnecessary contribution to landfill. | **Teleol. Conseq. (positive for environment):** Cindy’s decision means that there won’t be an unnecessary contribution to landfill. |
| **Teleological:**  **Negative consequences of main character’s behaviour to the environment / Code 0** | **10B.** **Scene:** It’s lunchtime so Cindy leaves the office and heads to a café with her colleagues. The café provides throwaway plastic cutlery (knives, forks etc.) to use, but Cindy normally brings her own metal, washable cutlery instead. Once she places her order, Cindy realises that she has left her cutlery at the office. The café is only a few minutes’ walk from the office, but it has started raining outside and she hasn’t brought an umbrella. | **10D. Scene:** It’s lunchtime so Cindy leaves the office and heads to a café with her colleagues. The café provides throwaway plastic cutlery (knives, forks etc.) to use, but Cindy normally brings her own metal, washable cutlery instead. Once she places her order, Cindy realises that she has left her cutlery at the office. The café is only a few minutes’ walk from the office, but it has started raining outside and she hasn’t brought an umbrella. |
|  | **Environmental case:** Cindy knows that the plastic cutlery goes to landfill sites and pollutes the environment, which is why she and her colleagues normally bring their own. | **Environmental case:** Cindy knows that the plastic cutlery goes to landfill sites and pollutes the environment, which is why she and her colleagues normally bring their own. |
|  | **Deont. Behaviour unethical:** Cindy decides that she doesn’t want to get wet and picks up some plastic cutlery. | **Deont. Behaviour ethical:** Cindy decides it doesn’t matter if she will get wet and heads back to the office to pick up her cutlery. |
|  | **Circumstances**. n/a | **Circumstances**. However, Cindy’s colleagues say that she is crazy for going out in the rain and does not let her go back to the office. Cindy uses the plastic cutlery instead. |
|  | **Teleol. Conseq. (negative for environment):** Cindy’s decision means that there will be an unnecessary contribution to landfill. | **Teleol. Conseq. (negative for environment):** The colleague’s decision means that there will be an unnecessary contribution to landfill. |

**Supplementary Table 1.** Shapiro-Wilk test results for normality assumptions.

| **Variable** | **Statistic** | **df** | ***p*** |
| --- | --- | --- | --- |
| **Dilemmas A, (Unethical, Positive), *N* = 141** | | | |
| Ethical Evaluation | .969 | 141 | .003 |
| Intention and Behaviour – Recycling | .833 | 141 | .000 |
| Intention and Behaviour – Composting | .907 | 141 | .000 |
| Intention and Behaviour – Transportation | .951 | 141 | .000 |
| Intention and Behaviour – Meat Consumpt. | .962 | 141 | .001 |
| Intention and Behaviour – Energy Use | .918 | 141 | .000 |
| Intention and Behaviour – Utensils | .854 | 141 | .000 |
|  |  |  |  |
| **Dilemmas B, (Unethical, Negative), *N* = 139** | | | |
| Ethical Evaluation | .981 | 139 | .046 |
| Intention and Behaviour – Recycling | .806 | 139 | .000 |
| Intention and Behaviour – Composting | .876 | 139 | .000 |
| Intention and Behaviour – Transportation | .957 | 139 | .000 |
| Intention and Behaviour – Meat Consumpt. | .945 | 139 | .000 |
| Intention and Behaviour – Energy Use | .918 | 139 | .000 |
| Intention and Behaviour – Utensils | .806 | 139 | .000 |
|  |  |  |  |
| **Dilemmas C, (Ethical, Positive), *N* = 136** | | | |
| Ethical Evaluation | .904 | 136 | .000 |
| Intention and Behaviour – Recycling | .849 | 136 | .000 |
| Intention and Behaviour – Composting | .893 | 136 | .000 |
| Intention and Behaviour – Transportation | .943 | 136 | .000 |
| Intention and Behaviour – Meat Consumpt. | .946 | 136 | .000 |
| Intention and Behaviour – Energy Use | .921 | 136 | .000 |
| Intention and Behaviour – Utensils | .820 | 136 | .000 |
|  |  |  |  |
| **Dilemmas D, (Ethical, Negative) *N* = 141** | | | |
| Ethical Evaluation | .939 | 141 | .000 |
| Intention and Behaviour – Recycling | .817 | 141 | .000 |
| Intention and Behaviour – Composting | .901 | 141 | .000 |
| Intention and Behaviour – Transportation | .963 | 141 | .001 |
| Intention and Behaviour – Meat Consumpt. | .956 | 141 | .000 |
| Intention and Behaviour – Energy Use | .901 | 141 | .000 |
| Intention and Behaviour – Utensils | .816 | 141 | .000 |

**Supplementary Table 2.** Means, standard deviations, and paired samples *t*-tests for intention and behaviour variables in dilemmas A, B, C, D.

| **Variable** | ***M***  **Intention** | ***SD***  **Intention** | ***M***  **Behaviour** | ***SD***  **Behaviour** | ***M* Difference** | **Cohen’s *d*** | ***t*** | ***df*** | ***p*** |
| --- | --- | --- | --- | --- | --- | --- | --- | --- | --- |
| **Dilemmas A** | | | | | | | | | |
| 1. Recycling | 6.03 | 1.39 | 5.02 | 1.82 | 1.02 | 1.40 | 8.63 | 140 | .000 |
| 2. Composting | 4.08 | 2.21 | 2.88 | 2.20 | 1.20 | 1.61 | 8.87 | 140 | .000 |
| 3. Transportation | 4.57 | 1.90 | 3.72 | 1.90 | .85 | 1.52 | 6.68 | 140 | .000 |
| 4. Meat Consumption | 4.52 | 1.86 | 3.75 | 1.85 | .77 | 1.49 | 6.11 | 140 | .000 |
| 5. Energy | 5.79 | 1.30 | 4.74 | 1.56 | 1.05 | 1.48 | 8.45 | 140 | .000 |
| 6. Utensils | 2.87 | 1.96 | 2.05 | 1.73 | .85 | 1.80 | 5.43 | 140 | .000 |
|  | | | | | | | | | |
| **Dilemmas B** | | | | | | | | | |
| 1. Recycling | 6.00 | 1.55 | 5.01 | 1.88 | .99 | 1.24 | 9.45 | 138 | .000 |
| 2. Composting | 3.73 | 2.34 | 2.47 | 2.08 | 1.27 | 1.77 | 8.45 | 138 | .000 |
| 3. Transportation | 4.36 | 2.00 | 3.24 | 1.82 | 1.12 | 1.81 | 7.33 | 138 | .000 |
| 4. Meat Consumption | 4.58 | 2.06 | 3.65 | 1.88 | .93 | 1.54 | 7.10 | 138 | .000 |
| 5. Energy | 5.78 | 1.32 | 4.70 | 1.76 | 1.09 | 1.49 | 8.61 | 138 | .000 |
| 6. Utensils | 2.59 | 1.98 | 1.60 | 1.31 | 1.00 | 1.93 | 6.03 | 138 | .000 |
|  | | | | | | | | | |
| **Variable** | ***M***  **Intention** | ***SD***  **Intention** | ***M***  **Behaviour** | ***SD***  **Behaviour** | ***M* Difference** | **Cohen’s *d*** | ***t*** | ***df*** | ***p*** |
| **Dilemmas C** | | | | | | | | | |
| 1. Recycling | 6.00 | 1.39 | 4.87 | 1.89 | 1.13 | 1.47 | 8.93 | 135 | .000 |
| 2. Composting | 3.68 | 2.16 | 2.60 | 2.01 | 1.08 | 1.55 | 8.13 | 135 | .000 |
| 3. Transportation | 4.42 | 2.03 | 3.43 | 1.91 | .99 | 1.73 | 6.68 | 135 | .000 |
| 4. Meat Consumption | 4.37 | 2.00 | 3.60 | 1.94 | .77 | 1.51 | 5.95 | 135 | .000 |
| 5. Energy | 5.76 | 1.36 | 4.46 | 1.73 | 1.31 | 1.46 | 10.42 | 135 | .000 |
| 6. Utensils | 2.54 | 1.78 | 1.89 | 1.72 | .65 | 1.74 | 4.34 | 135 | .000 |
|  | | | | | | | | | |
| **Dilemmas D** | | | | | | | | | |
| 1. Recycling | 6.09 | 1.42 | 4.96 | 1.98 | 1.12 | 1.44 | 9.25 | 140 | .000 |
| 2. Composting | 3.71 | 2.10 | 2.55 | 2.01 | 1.16 | 1.43 | 9.65 | 140 | .000 |
| 3. Transportation | 4.44 | 1.87 | 3.43 | 1.86 | 1.01 | 1.76 | 6.81 | 140 | .000 |
| 4. Meat Consumption | 4.68 | 1.88 | 3.76 | 1.80 | .92 | 1.47 | 7.47 | 140 | .000 |
| 5. Energy | 5.91 | 1.30 | 4.81 | 1.72 | 1.10 | 1.40 | 9.30 | 140 | .000 |
| 6. Utensils | 2.69 | 1.93 | 1.86 | 1.65 | .83 | 1.68 | 5.86 | 140 | .000 |
